# Major contribution of anaplerosis to inorganic carbon fixation in the dark ocean

**DOI:** 10.1038/s41561-026-02013-1

**Published:** 2026-06-15

**Authors:** C. Amano, U. Willhelm, T. Koch, T. Reinthaler, R. L. Hansman, E. Sintes, G. J. Herndl, J. M. González, F. Baltar

**Affiliations:** 1 Fungal and Biogeochemical Oceanography Group, Department of Functional and Evolutionary Ecology, University of Vienna, Vienna, Austria; 2 Microbial Oceanography Group, Department of Functional and Evolutionary Ecology, University of Vienna, Vienna, Austria; 3 Woods Hole Oceanographic Institution, Woods Hole, MA USA; 4 Instituto Español de Oceanografía-Consejo Superior de Investigaciones Científicas (IEO-CSIC), Centro Oceanográfico de Baleares, Palma de Mallorca, Spain; 5 Department of Marine Microbiology and Biogeochemistry, Royal Netherlands Institute for Sea Research (NIOZ), Texel, the Netherlands; 6 Department of Microbiology, University of La Laguna, La Laguna, Spain; 7 Fungal and Biogeochemical Oceanography Group, College of Oceanography and Ecological Science, Shanghai Ocean University, Shanghai, China; 8 Present Address: Faculty of Environmental Earth Science, Hokkaido University, Sapporo, Japan

**Keywords:** Carbon cycle, Marine biology, Microbial biooceanography, Microbial ecology

## Abstract

While CO_2_ fixation by photo- and chemolithoautotrophs is a central process of the global carbon cycle, many organisms also incorporate inorganic carbon into organic compounds through anaplerotic carbon fixation, a process that replenishes intermediates of central metabolic pathways. However, the active drivers and quantitative importance of anaplerotic carbon fixation in the oceanic carbon cycling remain poorly understood. Here, through analysis of global ocean multi-omics datasets, we identified widespread expression of enzymes involved in this process, especially phosphoenolpyruvate carboxylase. The heterotrophic bacterial genus *Alteromonas*, a globally distributed marine taxon lacking genes for autotrophic carbon fixation pathways, exhibited particularly high transcriptional and proteomic activity for this enzyme. Laboratory incubations confirmed that *Alteromonas* assimilated dissolved inorganic carbon (DIC) into biomass, with rates regulated by temperature and organic matter availability. Single-cell tracer analyses of the deep ocean microbial communities quantified *Alteromonas*’s contribution at about 17% of total dark DIC fixation (median; confidence interval, 10–28%), equivalent to a potential global flux of about 0.2 PgC yr^−1^. These results reveal substantial DIC fixation via anaplerosis, indicating that dark carbon fixation is partly supported by heterotrophic metabolism and modulated by environmental conditions, with responses that may differ from those of canonical autotrophic processes.

## Abstract (web summary)

While most newly formed organic carbon in the dark ocean is derived from chemolithoautotrophic microorganisms, 17% also comes from an alternative pathway called anaplerosis that is related to the tricarboxylic acid cycle, according to multi-omics datasets and single-cell analysis.

## Main

Carbon fixation plays a vital role in Earth’s biosphere. Marine photoautotrophs inhabiting the euphotic zone fix approximately 50 PgC yr^−1^ of CO_2_, accounting for roughly half of the Earth’s net primary productivity^1^. In the dark ocean, chemolithoautotrophic microorganisms also assimilate dissolved inorganic carbon (DIC), contributing an estimated ~1–3 PgC yr^−1^ to total carbon fixation^2,3^. Together, these processes convert inorganic carbon into organic forms, sustaining essential biogeochemical functions in the ocean^4^.

**Extended Data Fig. 1:**
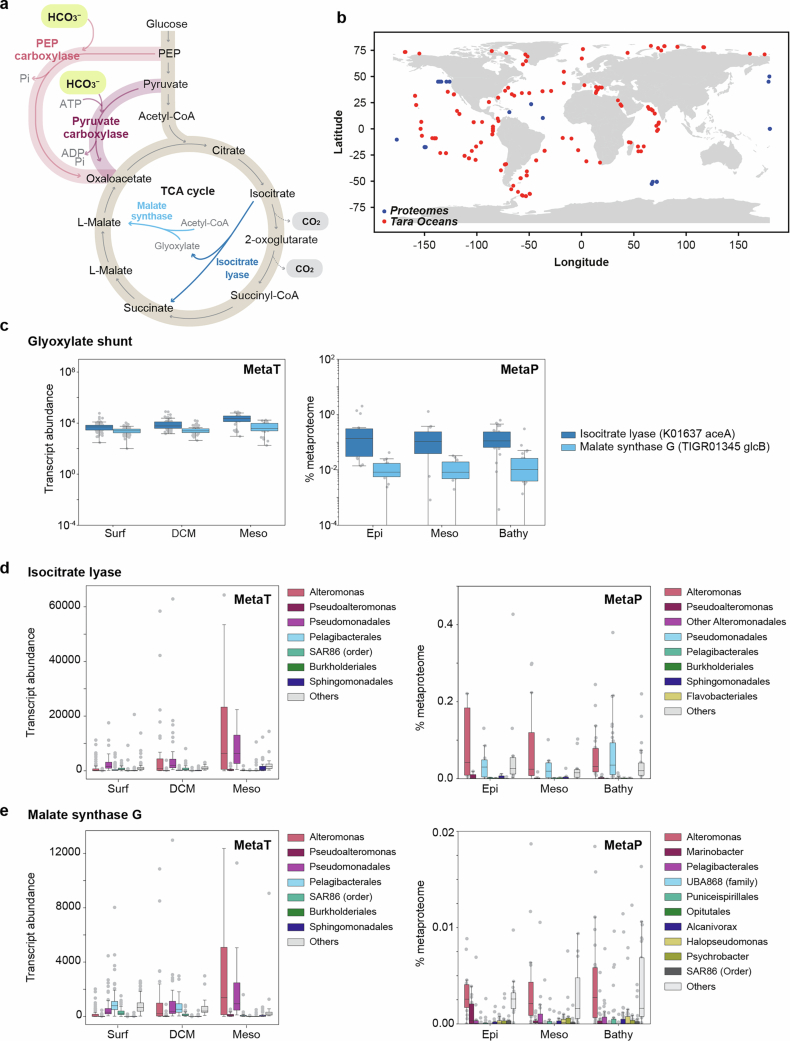
Sampling locations and abundance of glyoxylate shunt gene expression. (**a**) A schematic overview of anaplerotic pathways and TCA cycle. (**b**) Map of sampling location of the *Tara* Oceans metagenomic and -transcriptomic datasets (red) and metaproteomics datasets (blue) analyzed in this study. (**c**) Abundance of glyoxylate shunt genes in metatranscriptomic (MetaT; left, n = 187) and metaproteomic (MetaP; right, n = 61) datasets. (**d, e**) Main microbial taxa expressing glyoxylate shunt genes in the MetaT (left) and MetaP (right). Boxes represent distribution quartiles Q1 and Q3, while the horizontal line in the box represents the median. Whiskers represent the minimum and maximum values. Data from refs. ^16,49^.

Another process involved in DIC fixation, though often overlooked, is anaplerosis^5,6^. This process is ubiquitous in both autotrophic and heterotrophic organisms, including humans, and facilitated by enzymes, such as pyruvate carboxylase and phosphoenolpyruvate (PEP) carboxylases^7^. These anaplerotic enzymes catalyse the incorporation of DIC into oxaloacetate (Extended Data Fig. 1a), thereby replenishing tricarboxylic acid (TCA) cycle intermediates when they are depleted. Early studies from the 1960s showed that anaplerotic CO_2_ fixation generally contributes 7–8% of carbon biomass in heterotrophic bacteria grown on C_3_–C_6_ organic carbon sources (for example, glucose)^8^, while more recent studies suggest a general contribution of 1–8% to the carbon biomass in heterotrophs^5^. Despite its ubiquity, the ecological role and relevance of anaplerosis in the ocean remains largely understudied.

In the marine environment, anaplerotic DIC fixation has traditionally been considered a minor contributor to the total dark DIC fixation^3,9^. However, recent studies have reported relatively high DIC fixation potentially driven by heterotrophic microorganisms in sparse yet diverse oxygenated oceanic regions, including the coastal Pacific^10^, the Arctic surface^11^, the Mediterranean^12^ and sediments in the Gulf of California^13^. Moreover, recent work indicates that ammonia oxidizers contribute only modestly to dark DIC fixation^14^, supporting a potentially important role for heterotrophic metabolisms. However, the relative importance of anaplerotic enzymes, the active responsible taxa and the overall contribution of anaplerotic DIC fixation to total dark DIC fixation in the ocean remain poorly constrained, precluding the integration of this process into the oceanic carbon budget.

Here, we bridge this knowledge gap by first analysing global ocean multi-omics datasets, showing substantially high expression of anaplerotic enzymes and identifying the heterotrophic bacterium *Alteromonas*—which lacks canonical autotrophic pathways—as a key actor. We then validate this omics-derived indication through laboratory physiological measurements, demonstrating that an *Alteromonas* isolate actively fixes DIC in response to temperature and organic matter. To quantify its biogeochemical contribution, we apply microautoradiography combined with catalysed reporter deposition fluorescence in situ hybridization (MICRO–CARD–FISH) across oceanic regions and depths. By combining experimental and field-based constraints, we provide a first-order estimate of anaplerotic DIC fixation in the global ocean.

## Expression of DIC fixation genes in the global ocean

**Fig. 1:**
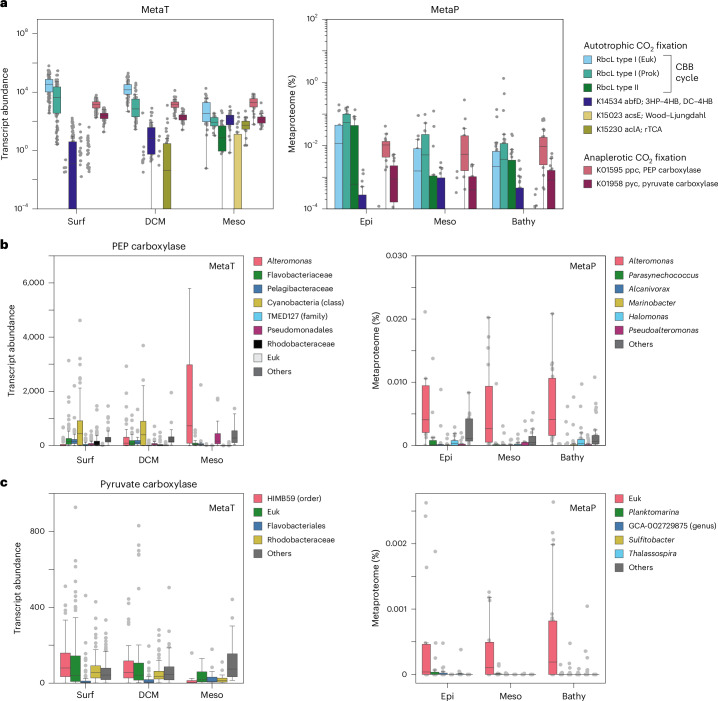
Expression of CO_2_-fixation genes by prokaryotes and eukaryotes in the global ocean, as analysed using metatranscriptomics and metaproteomics. **a**, Abundance of transcripts and protein expression of key marker genes for CO_2_-fixation pathways across different depth zones, determined by metatranscriptomic (MetaT; left, *n* = 187) and metaproteomic (MetaP; right, *n* = 61) analyses. Indicated KEGG KOs specific to each enzyme were used to quantify their contributions. HMMs targeting the RuBisCO large subunit (RbcL) type I and II^46^ and the ATP citrate lyase-α subunit (rTCA cycle) in *Nitrospina* (designed in this study) were created to improve annotations. **b**,**c**, Main microbial taxa dominating the metatranscriptomes (left) and metaproteomes (right) of anaplerotic enzymes PEP carboxylase (**b**) and pyruvate carboxylase (**c**). Transcriptomic estimates were performed using the methodology described in a previous study^46^ (see the Methods for more details) and are shown as the amount of gene transcription. The metaproteomics dataset, obtained from a prior study^16^, is shown as the presence of genes in the total metaproteome. Depth layers differ between datasets according to the original sampling depths. Boxes represent the interquartile range (Q1–Q3), with the central line indicating the median; whiskers denote the minimum and maximum values. Surf, surface (5–10 m); DCM, (20–200 m); Epi, epipelagic (<200m); Meso, mesopelagic (200–1,000 m); Bathy, bathypelagic (1,000–4,000 m); Euk, eukaryotes; Prok, prokaryotes.

The *Tara* Oceans metagenomic and metatranscriptomic dataset^15^ as well as a global ocean metaproteomic dataset^16^ were compiled and analysed by quantifying the abundance of transcripts and proteins of marker genes to assess the expression of anaplerotic CO_2_-fixation genes in the global ocean (Extended Data Fig. 1b). For comparison, we also examined marker genes of other DIC-fixation pathways, including RuBisCO for the Calvin–Benson–Bassham (CBB) cycle, and key enzymes of major chemolithoautotrophic processes such as the reverse TCA (rTCA) cycle and the 3-hydroxypropionate–4-hydroxybutyrate (3HP–4HB), dicarboxylate–4-hydroxybutyrate (DC–4HB) and Wood–Ljungdahl pathways. These pathways mediate most autotrophic CO_2_ assimilation^17^, converting inorganic carbon to the organic form via carboxylation^7^. Within anaplerotic DIC fixation pathways, PEP carboxylase dominated, exhibiting approximately tenfold-higher expression than pyruvate carboxylase (Fig. 1a). By contrast with the decline in RuBisCO expression with depth, PEP carboxylase remained consistently high throughout the water column in the metatranscriptomes, resulting in a higher relative contribution of PEP carboxylase expression below the deep chlorophyll maximum (DCM) layer (20–200 m; Tukey-adjusted comparison of below- versus above-DCM, *P* ≤ 0.013; Fig. 1a). Remarkably, the translation and expression of PEP carboxylase was higher than that of non-CBB autotrophic CO_2_-fixation pathways at all the depth layers in both the metatranscriptomes and metaproteomes (paired pathway contrasts, Tukey-adjusted *P* < 0.0001 for all comparisons; Fig. 1a). In addition, isocitrate lyase and malate synthase, key components of the glyoxylate shunt, were highly expressed (Extended Data Fig. 1c). This pathway bypasses CO_2_-producing steps of the TCA cycle, enabling cells to divert acetyl-CoA towards biosynthesis while minimizing carbon loss^18^ (Extended Data Fig. 1a). Their high expression alongside PEP carboxylase suggests active carbon replenishment through anaplerotic pathways in oceanic microorganisms.

While cyanobacteria dominated PEP carboxylase at the surface (5–10 m) and DCM in the metatranscriptome, their proteins were basically absent in the metaproteome. By contrast, *Alteromonas* exhibited high and consistently detectable PEP carboxylase throughout the water column, with particularly elevated levels in the metaproteome (Fig. 1b). The concurrent high expression of glyoxylate shunt genes (isocitrate lyase and malate synthase; Extended Data Fig. 1d,e) by *Alteromonas* is consistent with a carbon-conserving metabolic strategy that supports the replenishment of TCA cycle intermediates during heterotrophic growth. Notably, the lack of chemolithoautotrophic DIC fixation pathways as well as the pyruvate carboxylase gene in their genome is consistent with our observation that *Alteromonas* dominates all anaplerotic enzymes except pyruvate carboxylase (Fig. 1c); we therefore focused on *Alteromonas* as a model heterotroph for anaplerotic CO_2_ fixation.

**Extended Data Fig. 2:**
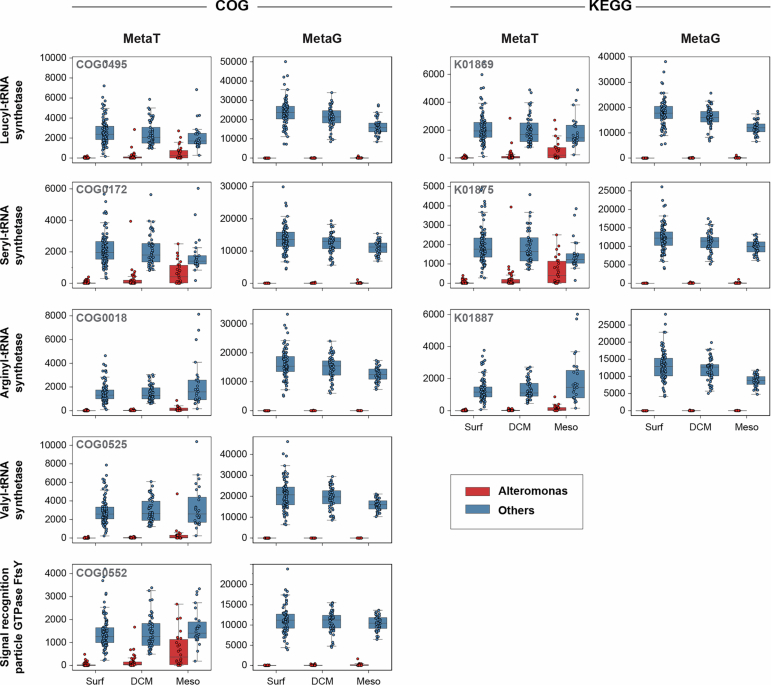
Contributions of single-copy housekeeping genes in the metagenome and metatranscriptome of the Tara Oceans dataset. The metagenomic (MetaG) and metatranscriptomic (MetaT) datasets were directly obtained from the abundance table in Salazar et al.^49^ (MetaT, n = 187; MetaG, n = 180). Contribution of house-keeping genes classified as *Alteromonas* was obtained for the 51 samples for which both metatranscriptome and metagenome were available (see Methods). The figure shows that *Alteromonas* (red) is low in abundance (MetaG) but high in activity (MetaT) compared to the other taxa (blue), with this trend being particularly pronounced in the mesopelagic waters. Lists of KO (left) and COGs (right) are derived from Sánchez et al.^78^. Boxes represent the distribution quartiles Q1 and Q3, with the horizontal line inside the box representing the median. Whiskers represent the minimum and maximum values.

Despite usually accounting for a small fraction of microbial communities (<1–3%)^19,20^, *Alteromonas* typically exhibit high growth rates^21^, reflecting a copiotrophic lifestyle^22^. Their high metabolic activity and organic matter-degradation capability^23^ are often associated with episodic organic matter inputs, such as phytoplankton blooms and marine snow^20,24^. Owing to their large cell size and copiotrophic nature, *Alteromonas* have been reported to disproportionately contribute to carbon cycling and ecosystem functioning^25^. Consistent with this, we found higher transcriptome-to-genome ratios for housekeeping and anaplerotic genes in *Alteromonas* than in other marine microorganisms (Extended Data Fig. 2), indicating elevated metabolic potential and transcriptional activity. A recent study reported that *Alteromonas*’s high metabolic activity despite their typically limited population size coincides with strong viral pressure, as evidenced by elevated viral markers and *Alteromonas*-derived proteins dominating the dissolved pool^16^, indicating that top-down control via viral lysis probably maintains *Alteromonas* populations in a highly active yet low-abundance state by rapidly recycling cells into the dissolved organic matter pool. Global metaomics analyses also show that *Alteromonas* dominates the metatranscriptomic and metaproteomic profiles of the primary enzymes responsible for dissolved organic phosphorus utilization, highlighting its broader biogeochemical relevance and extending its influence to the phosphorus cycle^26^. Therefore, the pronounced expression of PEP carboxylase and glyoxylate shunt genes by *Alteromonas* reflects its ecological strategy of sustaining high and versatile metabolism and rapid substrate turnover despite intense top-down control, thereby supporting its disproportionate role in ocean carbon and nutrient cycling.

Overall, this global ocean metatranscriptomic and metaproteomic analysis identified PEP carboxylase as the most highly expressed and produced anaplerotic enzyme, with expression levels comparable to, or even exceeding, those of autotrophic CO_2_-fixation pathways below the DCM layer (type III analysis of variance (ANOVA) *P* < 0.0001; Tukey-adjusted contrasts: MetaT, PEP carboxylase not lower than any CO_2_-fixation pathway, *P* > 0.05; MetaP, PEP carboxylase highest relative expression across pathways, majority of contrasts *P* < 0.001). *Alteromonas* showed consistently high PEP carboxylase expression across diverse regions, highlighting this genus as a compelling model taxon for deeper investigation of anaplerotic CO_2_ fixation by heterotrophic prokaryotes in the dark ocean.

## Anaplerotic DIC fixation by an *Alteromonas* representative

**Fig. 2:**
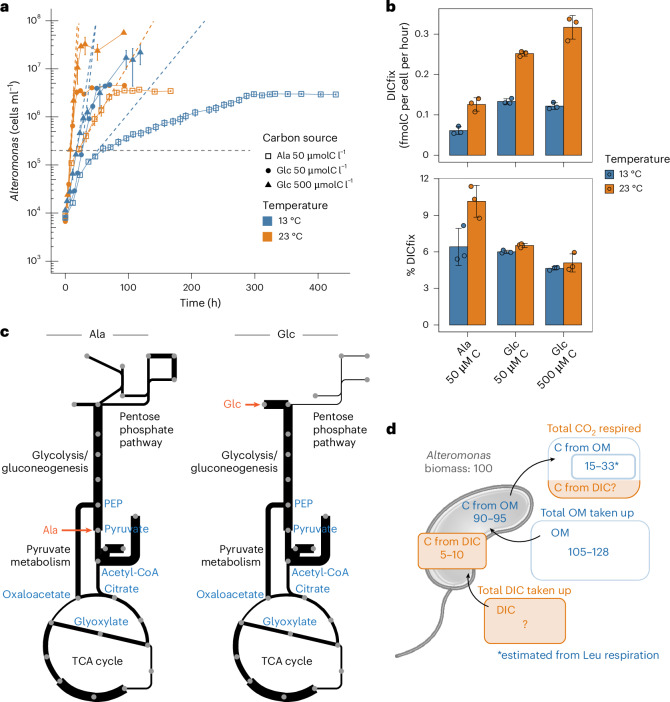
Anaplerotic carbon fixation by the *Alteromonas* strain under different organic carbon and temperature conditions. **a**, Growth curve of AltDE. Cells in early exponential phase (~2 × 10^5^ cells ml^−1^) indicated by the dashed line were used to measure cell-specific DIC fixation and cell-specific heterotrophic biomass production (Methods). Dashed lines show the slopes of growth (*μ* = ln 2/*g*) anchored to the cell abundances at the time of sampling. **b**, Cell-specific DIC fixation rates of AltDE (top); per cent contribution of DIC fixation to heterotrophic biomass production (% DICfix = cell-specific DIC fixation/cell-specific heterotrophic biomass production × 100 (bottom)). **c**, Flux distribution predicted by COBRApy using a genome-scale metabolic model for AltDE constrained with gene expression from a *Tara* Oceans metatranscriptome. Each node represents a compound in the TCA cycle, glycolysis or the pentose phosphate pathway, and the thickness of the lines connecting the nodes is proportional to the estimated carbon flow through the reactions. **d**, Conceptual flow diagram illustrating the carbon amount derived from DIC and organic matter to AltDE under the studied conditions, assuming an AltDE biomass of 100. Points and bars represent mean values; error bars indicate ±s.d. of *n* = 3. Ala, alanine; Glc, glucose, OM, organic matter.

To determine whether DIC assimilated via anaplerotic carbon fixation contributes to the biomass of *Alteromonas* cells and how it compares to heterotrophic biomass production, that is, the synthesis of bacterial biomass from organic precursors^27,28^ (here measured via ^14^C-leucine incorporation and respiration via ^14^CO_2_ release), we conducted experiments under controlled laboratory conditions using the cultured representative *Alteromonas mediterranea* (deep ecotype, further referred to as AltDE)^29,30^. As previous studies indicated that the quality of organic carbon sources might influence prokaryotic anaplerotic carbon fixation^31–33^, culture experiments were conducted with alanine (Ala)—a major amino acid in the ocean^34^—and glucose (Glc)—a representative and common sugar—with the baseline condition set at 50 µmolC l^−1^ and 13 °C. To examine the effects of carbon enrichment and warming, we included a high Glc concentration (500 µmolC l^−1^) representing coastal phytoplankton bloom conditions^35^ and an elevated temperature treatment (23 °C). DIC fixation was determined by incubating cultures with ^14^C-bicarbonate and measuring assimilation into biomass, and cell-specific DIC rates were calculated by normalizing DIC fixation to cell abundance during the exponential growth (Fig. 2a). To examine how anaplerotic CO_2_ fixation is integrated into the central metabolism of AltDE, we also reconstructed a genome-scale metabolic model of AltDE and performed flux balance analysis and simulated flux distribution on Glc and Ala as the sole organic carbon source.

**Extended Data Fig. 3:**
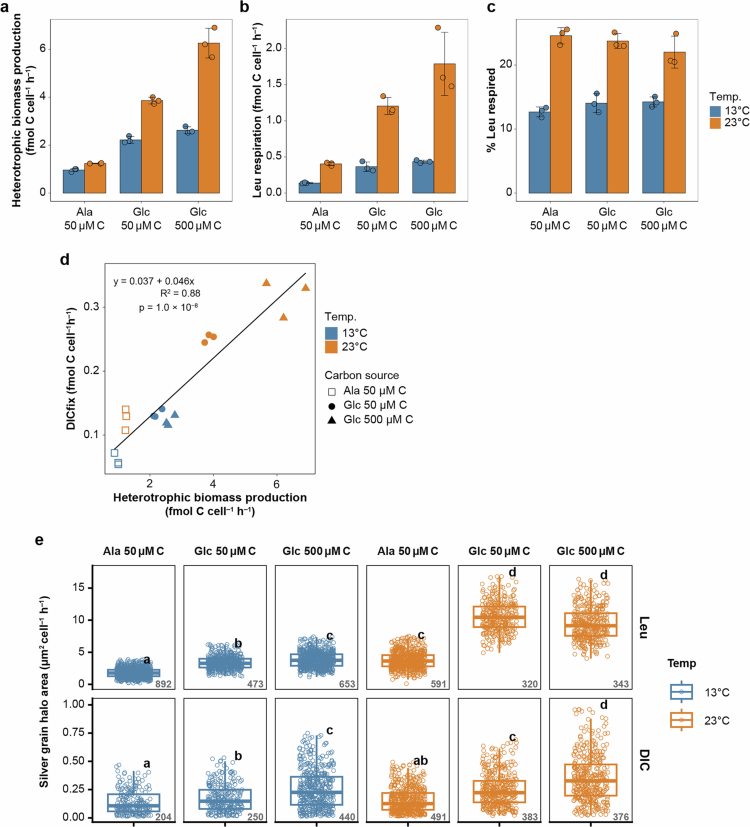
Cell-specific heterotrophic activity of Alteromonas mediterranea determined by bulk and single-cell measurements under different organic carbon sources and temperatures. Cell-specific heterotrophic biomass production (**a**) and leucine respiration (**b**) rates of *Alteromonas mediterranea* (AltDE). (**c**) Percentage contribution of leucine respiration to total leucine uptake, calculated as cell-specific Leu respiration divided by (cell-specific Leu incorporation + cell-specific Leu respiration), expressed as percentages. Bars indicate mean values; error bars represent ± s.d. of n = 3. (**d**) Relationship between cell-specific heterotrophic biomass production and cell-specific DIC fixation. (**e**) Silver grain halo sizes around individual AltDE cells from microautoradiography, indicative of their activity levels^37^. Numbers in each panel indicate sample size (n). Letters next to the boxplots represent results from a post hoc Dunn test following Kruskal-Wallis multiple comparisons, with Leu and DIC samples analyzed separately. Boxes represent the interquartile range (Q1–Q3) with the center lines indicating medians; whiskers extend to 1.5 × IQR. Glc: glucose, Ala: alanine.

**Extended Data Fig. 4:**
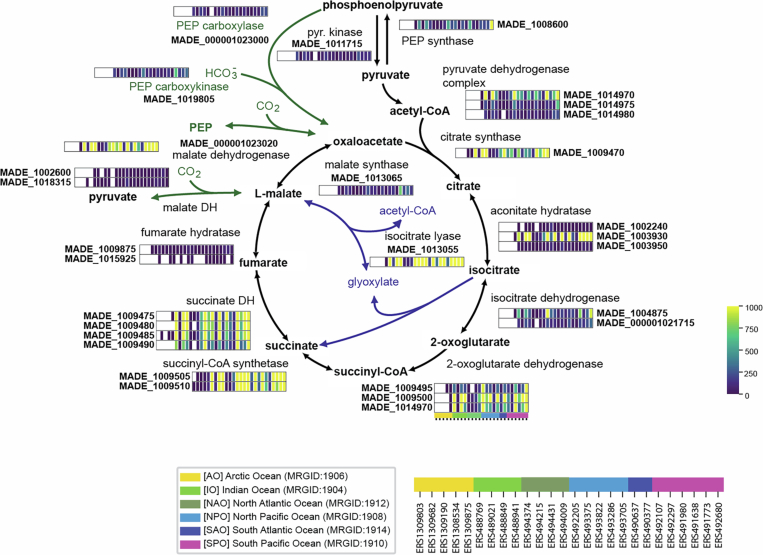
Fragment recruitment of environmental reads to Alteromonas mediterranea (AltDE) genes that encode the enzymes in the TCA cycle, anaplerotic and glyoxylate shunt. The *Tara* Oceans mesopelagic metatranscriptome samples were aligned with BBMap against AltDE ORFs, as described in Methods. The heatmaps show the average coverage for each ORF for each of the samples. The labels next to the heat maps are the KEGG entries for the ORF. The samples are sorted by the oceanic region as colored in the legend with their European Nucleotide Archive (ENA) ID’s.

Our laboratory experiments empirically demonstrate that *Alteromonas* assimilates DIC into biomass during heterotrophic growth, with rates modulated by temperature and broadly coupled to respiration and carbon turnover (Fig. 2 and Extended Data Fig. 3; see the Supplementary Discussion for details). When expressing DIC fixation as a proportion of heterotrophic biomass production (% DICfix = cell-specific DIC fixation/cell-specific heterotrophic biomass production), DIC fixation contributed 4.6–10.2% (mean ± s.d., 6.5% ± 1.9%, *n* = 6; Fig. 2b), consistent with the earlier studies on other bacteria from the 1960s^8^. DIC fixation was consistently lower than respiratory carbon losses across all treatments (Fig. 2d and Extended Data Fig. 3). The lack of response to substrate enrichment at 13 °C suggests that assimilation is kinetically constrained under lower temperatures, which might be particularly relevant in the deep sea, where temperatures typically range between 2 and 4 °C, including relatively warm deep waters such as those of the Mediterranean Sea (~13 °C). Furthermore, our flux balance modelling of carbon flow indicates that anaplerotic carbon fixation and the glyoxylate shunt jointly sustain the replenishment of the TCA cycle in AltDE (Fig. 2c and Extended Data Fig. 4; see the Supplementary Discussion for details). Together, these results support DIC fixation as an integral, physiologically regulated component of *Alteromonas* central metabolism, essential for replenishing TCA-cycle intermediates and sustaining high heterotrophic flux.

## Putative anaplerotic DIC fixation by *Alteromonas* in the pelagic ocean

**Fig. 3:**
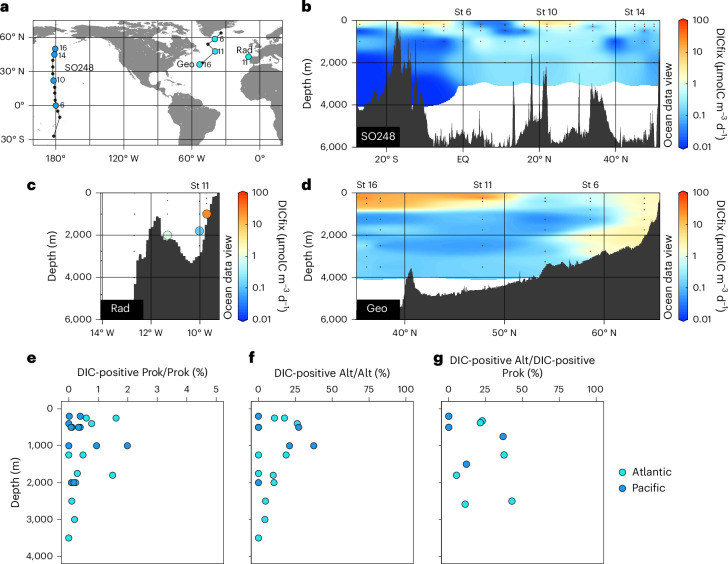
Bulk dark and putative anaplerotic DIC fixation by *Alteromonas* in the North Atlantic and the Pacific Ocean. **a**, Sampling locations occupied during the three cruises. Larger symbols with station (St) numbers indicate locations where MICRO–CARD–FISH was additionally performed, with different colours representing different oceanic basins. **b**–**d**, Sectional views of bulk dark DIC fixation in the Pacific (SO248) (**b**), off the Iberian Margin (Rad) (**c**) and in the North Atlantic Ocean (Geo) (**d**). Rates are shown on a log scale. Bathymetry from the GEBCO 2023 global grid^47^. **e**,**f**, Relative abundance of DIC-positive prokaryotes (**e**) and DIC-positive *Alteromonas* (Alt, **f**), shown as percentages of total prokaryotes and *Alteromonas*, respectively. DIC-positive cells refer to those that have taken up the tracer ^14^C-bicarbonate, as determined by MICRO–CARD–FISH. For each sample, >1,000 DAPI-stained cells were examined. **g**, Contribution of *Alteromonas* to the total prokaryotic dark DIC fixation. As the abundance of DIC-assimilating cells was low, values within each water mass were pooled, and only those containing ≥5 DIC-positive prokaryotes were kept. Values represent the proportion of DIC-positive *Alteromonas* among all DIC-positive prokaryotes in each water mass. Points are plotted at the average sampling depth for each water mass (Extended Data Table 1). Coastline data in **a** from the Global Self-consistent, Hierarchical, High-resolution Geography (GSHHG) database^48^.

**Extended Data Fig. 5:**
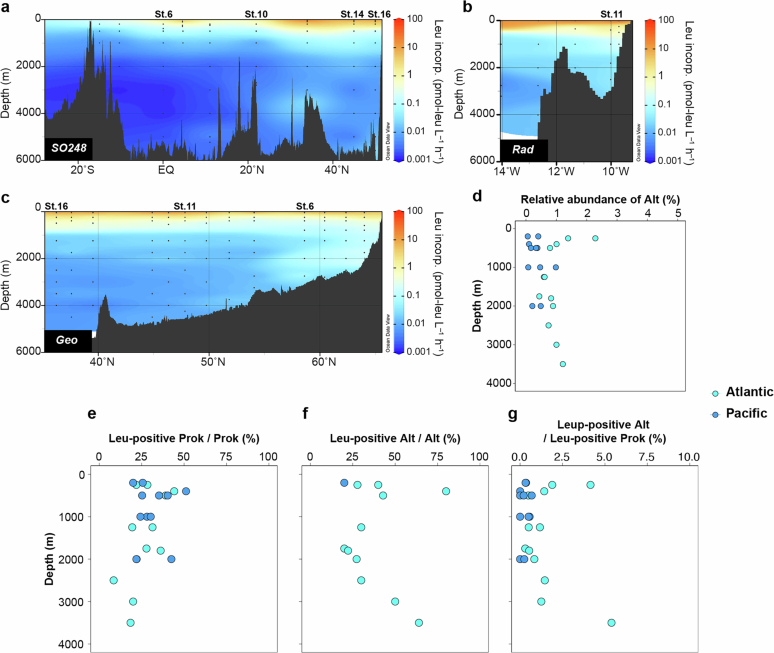
Distribution of prokaryotic heterotrophic activity, shown as leucine incorporation rates, in the Pacific and Atlantic Ocean. (**a–c**) Transect views of leucine incorporation rates (log scale) during SO248 in the Pacific (**a**), Rad (**b**) and Geo (**c**) cruises in the Atlantic Ocean (refer to Fig. 3a). Bathymetry from the GEBCO 2023 global grid^47^. (**d**) Mean relative abundance of *Alteromonas* in the MICRO-CARD-FISH samples for DIC and leucine assays. The consistently low relative abundance of *Alteromonas* indicates that there was no copiotrophic growth of *Alteromonas* at the cell abundance level during the incubations in these samples. (**e, f**) Relative abundance of leucine-positive prokaryotes and leucine-positive *Alteromonas* to total prokaryotes and total *Alteromonas*, respectively, as determined by MICRO-CARD-FISH. Only samples with a denominator of ≥5 cells are presented. (**g**) Relative abundance of leucine-positive *Alteromonas* to leucine-positive prokaryotes. Alt, Alteromonas; Prok, Prokaryotes.

To estimate anaplerotic DIC fixation by *Alteromonas* in the global ocean, we analysed samples incubated with ^14^C-bicarbonate to quantify DIC assimilation into biomass at both the bulk and single-cell level during three cruises in the Atlantic and Pacific Ocean (Geotraces (Geo), RadProf 2015 (Rad) and PacGeoBac (SO248); ≥200 m; Fig. 3a) and compared it with heterotrophic activity, measured in parallel incubations based on ^3^H-leucine incorporation (Methods). Bulk dark DIC fixation was generally high in the shallower waters, decreasing with depth (Fig. 3b–d), consistent with heterotrophic activity^36^ (Extended Data Fig. 5a–c). In the Pacific, dark DIC fixation rates were the highest (>15 µmolC m^−3^ d^−1^) at ~200 m in the South and North Pacific subtropical gyres, and low ( < 0.5 µmolC m^−3^ d^−1^) at bathypelagic depths (1,000–4,000 m; Fig. 3b). In the Atlantic, high dark DIC-fixation rates (>30 µmolC m^−3^ d^−1^) were found at 250 m from 50° N southwards, and relatively high rates (~2 µmolC m^−3^ d^−1^) were observed at the bathypelagic depths of the northern stations (Fig. 3d), probably owing to the formation of young deep-water masses^3^. However, these bulk dark DIC-fixation measurements, obtained using the commonly used standard protocol^9^, include DIC fixation from both chemolithoautotrophic and anaplerotic processes and cannot distinguish the relative proportion of each process.

We applied microautoradiography to enumerate ^14^C-bicarbonate uptake at the single-cell level, where intracellular tracer incorporation is visualized as a silver-grain halo around cells proportional to cell-specific DIC uptake^37^, and combined this with CARD–FISH to identify *Alteromonas*, showing high PEP carboxylase expression in global metaomics analyses (Fig. 1). Consistent with previous observations^9^, only 0–2% of all prokaryotes below the epipelagic layers (0–200 m) were DIC positive (Fig. 3e), reflecting the low abundance of cells assimilating DIC at detectable levels with microautoradiography in the dark ocean. Across all samples, *Alteromonas* represented ~1% of total prokaryotes on average (*n* = 23; Extended Data Fig. 5d) and within this taxon, 0–25% were DIC positive (Fig. 3f). In the Pacific, the contribution of *Alteromonas* to dark DIC fixation, expressed as the percentage of DIC-positive prokaryotic cells that are identified as *Alteromonas*, was generally low, with values between 0% and 37% (median, 6%, *n* = 4; Fig. 3g and Extended Data Table 1). In the North Atlantic, the contribution of *Alteromonas* to the dark DIC fixation was higher than in the Pacific, ranging from 5% to 43% (median, 22%, *n* = 6; Fig. 3g and Extended Data Table 1). Although the number of samples was small, these data indicate that *Alteromonas* forms a consistently detectable component of the dark DIC-fixing community across major ocean basins.

MICRO–CARD–FISH analyses also characterized the heterotrophic activity of prokaryotes. Across all prokaryotes, 29% ± 10% (mean ± s.d., *n* = 23; Extended Data Fig. 5e) of cells were heterotrophically active (that is, those incorporating ^3^H-leucine). Within *Alteromonas*, 40% ± 28% of cells incorporated leucine (Extended Data Fig. 5f), indicating that *Alteromonas* cells were more frequently active than the average heterotrophic marine prokaryote, despite their low relative abundance. This observation is further supported by the relatively higher expression of housekeeping genes of *Alteromonas* compared with other prokaryotes (Extended Data Fig. 2).

Overall, *Alteromonas* accounted for 0–43% of DIC-positive prokaryotic cells (median 17%, 95% bootstrap confidence interval (CI): 10–28%; *n* = 10; Extended Data Table 1), corresponding to up to 43% of dark DIC fixation, assuming comparable per-cell DIC fixation rates among DIC-positive taxa (Methods). To place this contribution in a global context, we provide a first-order extrapolation of potential anaplerotic DIC fixation by *Alteromonas*. Assuming 1 mmolC m^−2^ d^−1^ of depth-integrated dark DIC-fixation rate from a regression model in the North Atlantic^3^, scaling this to the global dark ocean (3.08 × 10^14^ m^2^ of mesopelagic surface area^38^) and applying the observed fraction of DIC-positive cells identified as *Alteromonas* yields a potential flux of up to 0.6 PgC yr^−1^ (median, 0.2 PgC yr^−1^). Using the 95% CI of DIC-positive cell fractions (10–28%) results in a corresponding range of 0.1–0.4 PgC yr^−1^. As this calculation is based on cell counts and does not include contributions from other heterotrophic taxa carrying out anaplerotic DIC fixation, it should be regarded as a conservative estimate. This magnitude is comparable to estimates of nitrification-driven DIC fixation (~0.1–1 PgC yr^−1^)^2,39^ and falls within the range of previously reported heterotrophic DIC fixation for marine and freshwater systems (0.02–4 PgC yr^−1^) indirectly estimated from heterotrophic biomass production^5^. Although modest relative to sinking particle export (~10 PgC yr^−1^)^40^, this pathway is currently not included in carbon cycle estimations and models.

Furthermore, following the logic of Sorokin’s original analysis^41,42^, we integrated dark DIC-fixation rates below the epipelagic layer in the North Atlantic (Geo) and North Pacific (SO248) and used these rates to infer the amount of organic carbon required to sustain heterotrophic communities. Because ~10% of heterotrophic biomass is derived from anaplerotic DIC fixation (Fig. 2), the measured DIC-fixation rates allow a back-calculation of the corresponding organic-carbon requirement (Methods). Applying this approach yields an estimated organic carbon requirement of 1.2 ± 0.6 PgC yr^−1^ (mean ± s.d., *n* = 10) for the North Pacific, a value comparable to published estimates of North Pacific export production (~1.2 PgC yr^−1^)^43^. By contrast, although in the same order, the North Atlantic requirement (5.2 ± 3.8 PgC yr^−1^, *n* = 6) exceeds reported export flux estimates (0.5–2 PgC yr^−1^)^44^ by a factor of ~3 or more. This apparent imbalance in the North Atlantic may reflect a combination of uncertainties in basin-scale extrapolation, large station-to-station variability and additional carbon sources such as fresh dissolved organic carbon (DOC) or suspended particulate organic carbon supplied from deep-water formation regions, as well as seasonal variability affecting demand and export estimates^45^. Given these uncertainties, the values should be viewed as approximate yet they remarkably highlight the potential carbon-supply constraints associated with heterotrophic anaplerosis at basin scales.

Anaplerosis is a conserved component of central metabolism yet its quantification in natural oceanic communities has been hindered by co-occurring autotrophic DIC fixation and dilution of tracer uptake by the vast ambient DIC pool. By combining taxon-targeted single-cell microautoradiography with metaomics and culture experiments, we identify ubiquitous heterotrophic lineages such as *Alteromonas* whose anaplerotic activity is sufficiently high to be detected and quantified relative to the broader community. The observed temperature sensitivity of anaplerotic DIC fixation in *Alteromonas* further suggests that this process may respond to ocean warming. This work thus moves anaplerosis from a cellular biochemical function to a recognized, quantitatively constrained component of the marine carbon cycle, refining our view of the carbon sources that sustain heterotrophic life in the dark ocean.

## Methods

### Quantification of gene expression in the global ocean

Key genes for carbon fixation and anaplerotic pathways were identified in the Ocean Microbial Reference Gene Catalog (OM-RGC.v2) to quantify their expression and abundance in the 187 metatranscriptomes and 180 metagenomes from the *Tara* Ocean expedition, corresponding to 109 stations^49^. The OM-RGC.v2 sequences, retrieved from https://www.ocean-microbiome.org, were translated with Prodigal version 2.6.3 (ref. ^50^). OM-RGC.v2 peptide sequences were placed on a reference tree for each predicted enzyme to assign a taxonomic label using previously described methods^46^, with details provided below.

The reference phylogenetic trees were constructed with a set of prokaryotic marine genomes from the previous study^46^, in addition to peptides from the Global Ocean Microbiome Catalogue genomes (43,191 genomes)^51^ and peptides from the eukaryotic genomes in EukProt proteins, version 3 (ref. ^52^). Prokaryotic genomes were classified with the GTDB Toolkit (GTDB-Tk) version 2.3.2 and database release 214 (ref. ^53^) to assign a taxonomic label to the peptides from the genome database. To make the reference phylogenetic trees for each gene, a hidden Markov model (HMM) profile was used to search for peptides in the genome database. RuBisCO type I and II were searched for following the previous study^46^. Acetyl-CoA/propionyl-CoA carboxylase is a common enzyme in both the 3-hydroxypropionate bicycle and the 3HP–4HB cycle in autotrophic archaea; corresponding peptides in Archaea were identified with K14534 using HMMER version 3.3.2 (ref. ^54^) and the adaptive score threshold^55^. Likewise, the reductive acetyl-CoA pathway (Wood–Ljungdahl pathway) was quantified using K15023, specific for 5-methyltetrahydrofolate corrinoid–iron sulfur protein methyltransferase. As very few hits were found for the key enzyme in the rTCA cycle, ATP citrate lyase-α subunit (K15230), which does not target *Nitrospina*, an HMM profile was designed for the homologue in *Nitrospina*. PEP carboxylase, pyruvate carboxylase and isocitrate lyase were detected with K01595, K01958 and K01637, respectively, also using their adaptive score thresholds. The two forms of the second enzyme in the glyoxylate shunt malate synthase, were detected with TIGR01344 (malate synthase A) and TIGR01345 (malate synthase G) using HMMER and the gathering score as threshold search for each. Here, we present malate synthase G, as it exhibited an expression level an order of magnitude higher than that of malate synthase A. Peptide selection, phylogenetic reconstruction and taxonomic assignment were performed using BLASTP, HMM-based searches and phylogenetic placement (see Supplementary Note 1 for full details).

Once the environmental peptides were functionally and taxonomically classified, the contribution of the taxa to the expression of the marker genes in the global ocean was quantified using the OM-RGC.v2 table of abundances at https://www.ocean-microbiome.org for the metatranscriptome and metagenomes in the *Tara* Ocean database for each sample^15^. Gene abundance was normalized by gene length and relative to housekeeping genes as explained in Kultima et al.^56^ and Sunagawa et al.^15^. As the *Tara* Ocean gene catalogue included a functional profile annotated by the Kyoto Encyclopedia of Genes and Genomes (KEGG) and Clusters of Orthologous Genes (COG), as well as taxonomic annotation, the contribution of housekeeping genes classified as *Alteromonas* was obtained for the 51 samples for which both metatranscriptome and metagenome were available.

Likewise, the enzymes were also predicted the same way and quantified in the metaproteomic study by Zhao et al.^16^ using their table of abundances associated with each peptide and sample, available in ref. ^57^. For this last set of environmental sequences, the peptides were normalized instead as a percentage relative to the rest of peptides in each sample. The average per cent identity between the classified peptides and those closest in the reference phylogenetic tree was above 95% in all cases, for both *Tara* Ocean and metaproteome peptides (see Supplementary Fig. 1 as an example with the metaproteome study). To reduce the number of sequences to a set of representatives, both the metagenomes and metatranscriptomes from the *Tara* Oceans dataset and the metaproteome sequences were clustered at 95% identity as detailed in the respective studies.

To evaluate the robustness of our quantification approach of gene expression, we compared our results with those obtained using a previously published gene quantification method applied to the same *Tara* Oceans dataset^49^. The two approaches yielded similar depth-dependent patterns of *Alteromonas* anaplerotic gene abundance (Supplementary Fig. 2), confirming the consistency of our quantification results. Our phylogeny-guided approach was required to correctly identify protein families that include paralogue sequences with functions not related to the pathway of interest across the enzymes examined (for example, RbcL type I and II)^46^. In addition, together with the use of newly created HMMs, and supported by several validation steps implemented throughout the pipeline, this approach enabled the detection and validation of lineage-specific homologues that are not targeted by the previous annotation profiles for certain pathways (for example, ATP citrate lyase in *Nitrospina*), thereby providing increased functional specificity and taxonomic resolution and reducing false-positive counts.

### Metabolic network reconstruction and model refinement

A draft metabolic model was built using the KBase Narrative Interface (www.kbase.us), which supports the reconstruction of metabolic models in microorganisms based on functional protein annotations. The genome of AltDE was re-annotated with RASTtk, version 1.073. Model reconstruction was performed with OMEGGA, followed by KBase’s automated gap-filling algorithm to improve network completeness. The resulting sbml file was downloaded and run on COBRApy version 0.29.1 for downstream simulations. The model was constrained using the metatranscriptome from one *Tara* Oceans sample (PANGEA sample ID TARA_B100000071; ref. ^58^) at latitude 19.0351° N, longitude 64.5638° E (Indian Ocean) and 340-m depth. The metatranscriptome reads were first processed as described in Baltar et al.^46^. Reads were aligned to the AltDE open reading frames (ORFs) using BBMap version 39.13 (ref. ^59^) with a minimum alignment identity threshold of 90% and allowing ambiguous reads to map to all possible locations. Coverage statistics (covstats) were generated for the AltDE metabolic model constraints. The medium was chosen to permit aerobic growth on alanine and glucose (Supplementary Table 1). The resulting carbon flows through each reaction were visualized in iPath3.0 (ref. ^60^).

To generate Extended Data Fig. 4, *Tara* Ocean metatranscriptome reads were first processed as described in Baltar et al.^46^. Reads were aligned to the AltDE ORFs using BBMap version 39.13 (ref. ^59^), applying a minimum alignment identity threshold of 90% and permitting ambiguous reads to map to all possible loci. Gene annotation was based on the entry at the KEGG database under organism code amc (ref. ^61^). Coverage statistics (covstats) were used to calculate the average number of reads covering each base in each ORF.

### Culture experiment with *A. mediterranea*

AltDE (Adriatic-2) was obtained from the Leibniz Institute DSMZ-German Collection of Microorganisms and Cell Cultures (ref. ^62^) and cultured for several generations in 0.2 µm filtered artificial seawater (ASW; Aquil* Medium, Bigelow). The ASW was supplemented with glucose (500 µMC) as a carbon source; NH_4_Cl and NaH_2_PO_4_·H_2_O as nitrogen and phosphorus source, respectively; and cyanocobalamin (0.037 nM) as a vitamin source. Cells in the late exponential phase were stored at −80 °C with 25% glycerol until the incubation experiments. For incubations, AltDE was cultured in ASW with either glucose (D-glucose; G8270, Sigma-Aldrich) or alanine (L-alanine; A7627, Sigma-Aldrich) as carbon sources, each at a concentration of 50 µMC. Two incubation temperatures, 13 °C and an elevated temperature of 23 °C, were chosen to assess DIC fixation by AltDE. These substrate concentrations and the 13 °C incubation were selected to reflect typical carbon concentrations and temperatures in the meso- and bathypelagic depths in the Mediterranean Sea^63^. To investigate the impact of increased carbon concentrations, an additional higher glucose treatment was introduced (500 µmolC l^−^^1^), reflecting the potentially elevated DOC concentration observed during coastal surface events, such as phytoplankton blooms^35^. For all media, the C:N:P ratio was maintained at 50:10:1 to ensure no limitations of nitrogen and phosphorus in any of the incubations^64,65^. Before the assays, the strain was pre-incubated in the corresponding media for more than three generations, and cells from the early exponential phase (~2 × 10^5^ cells ml^−1^) were used for the subsequent assays. These incubations were carried out in agitated 500-ml Erlenmeyer flasks with air-permeable silicone stoppers. All equipment was sterilized either by autoclaving or by combustion at 450 °C for 4 h.

Measurements included leucine incorporation and respiration, cell abundance, amino acid concentrations and cellular carbon content, which were used to estimate the contribution of anaplerotic DIC fixation. Detailed methods and calculations are provided in Supplementary Note 2.

### Collection of environmental samples

Sampling was carried out in the North Atlantic and the Pacific Ocean during three cruises: Geotraces (Geo, 2011), RadProf 2015 (Rad, 2015) and PacGeoBac (SO248, 2016) aboard research vessel (R/V) *Pelagia*, R/V *Ramon Margalef* and R/V *SONNE*, respectively (Fig. 3a). Seawater was collected from depths ranging between 250 and 3,500 m (Geo), 1,000 and 2,000 m (Rad) and 200 and 2,000 m (SO248) from a total of 23 stations. Epipelagic waters were not sampled for dark DIC-fixation measurements to avoid interference from photoautotrophs. The water was transferred from the Niskin bottles mounted on a conductivity, temperature, depth (CTD) rosette sampler into acid-washed 100–500-ml polycarbonate bottles for analysis of dark DIC fixation, leucine incorporation, prokaryotic abundance and MICRO–CARD–FISH. All samples were processed immediately for their respective analyses. Detailed information on the environmental parameters can be found at https://www.geotraces.org (Geo) and ref. ^66^ (SO248).

### Bulk dark DIC fixation

Culture samples of the AltDE strain and seawater samples from the three cruises were incubated in the dark with ^14^C-bicarbonate (specific activity, 50–60 mCi mmol^−1^, ARC0138B, Hartman Analytic; dissolved in sterile water adjusted to pH 9). For the AltDE experiment, ^14^C-bicarbonate was added to a final concentration of 8 µCi per sample and incubated for 4 h following the determination of their kinetics (Supplementary Fig. 3). For the environmental samples, incubation was conducted at in situ temperature ±1 °C with ^14^C-bicarbonate at concentrations of 50 or 100 µCi per sample for 2–3 days, following the previous studies^3,9^. For each depth, five aliquots were prepared: two were fixed immediately with 0.2 µm filtered formaldehyde at a final concentration of 2% (formaldehyde-killed blanks); all were then spiked with ^14^C-bicarbonate and, after incubation, the three live ones were fixed. For AltDE cultures, each biological replicate was divided into one blank and two live aliquots, with blanks fixed before tracer addition. All the formaldehyde fixed samples were then filtered onto polycarbonate filter (25-mm diameter, 0.2-µm pore size) and rinsed twice with 0.2 µm filtered seawater. The filters were exposed to HCl fumes overnight to remove ^14^C-bicarbonate not incorporated into the cells and subsequently placed into scintillation vials. After adding scintillation cocktail (Filter-Count, Canberra-Packard/PerkinElmer) to the vials, disintegrations per minute (DPMs) were measured using a scintillation counter (Tri-Carb, PerkinElmer). The mean blank DPM was then subtracted from the mean sample DPM to determine DIC fixation rates.

### Leucine incorporation

Leucine incorporation rate, a proxy of prokaryotic heterotrophic production^67–69^, was determined according to previous studies^3,9^. Seawater samples from the cruises were incubated at in situ temperature ± 1 °C in the dark with ^3^H-leucine (L-[3,4,5-^3^H]-leucine; specific acivity, 120 Ci mmol^−1^; final concentrations, 5 or 10 nM) for 2 h to 1 day, depending on the expected activity of the samples. After incubation, samples were fixed and filtered using the same procedure as for dark DIC fixation measurements. Filters were subsequently rinsed twice with 5% ice-cold trichloroacetic acid. The filters were placed in scintillation vials with scintillation cocktail, and the DPMs were measured using a scintillation counter (see above). It should be noted that leucine incorporation can be a proxy rather than strictly ambient prokaryotic production, as the addition of exogenous leucine may affect the availability of labile DOC, especially under substrate-limited conditions such as in oligotrophic waters. Nevertheless, the ^3^H-leucine approach is widely used, and the resulting values are comparable across depths and stations in this study.

### MICRO–CARD–FISH

Water samples from the AltDE cultures (10 ml) and the pelagic ocean samples (20–80 ml), previously incubated with either ^3^H or ^14^C-leucine or ^14^C-bicarbonate (as described above), were filtered onto 0.2-µm polycarbonate filters (25-mm filter diameter, GTTP, Millipore). The filters were rinsed twice with MQ water and stored at −20 °C until further analysis. Substrate uptake by *Alteromonas* collected on the filters was determined using MICRO–CARD–FISH^37,70^. Hybridization was conducted using a horseradish-peroxidase (HRP) labelled oligonucleotide probe targeting *Alteromonas/Colwellia* (Alt1413^71^) in a hybridization buffer with 55% formamide. Cell walls were permeabilized with lysozyme (10 mg ml^−1^), and amplification was carried out at 46 °C for 15 min using a 1:100 of tyramide-Alexa488 (1 mg ml^−1^). HRP-probes targeting most bacteria (EUB338^72^) and unspecific antisense control (NON388^73^) served as positive and negative controls, respectively. The Alt1413 probe was validated on the AltDE monoculture before environmental application, where it yielded clear, bright hybridization signals and detected all of the AltDE cells. For microautoradiography, previously hybridized cells were exposed to photographic emulsion (Nuclear Emulsion Type K5, ILFORD) at 4 °C for 2 weeks for ^3^H-leucine samples and 1 month for ^14^C-bicarbonate samples, on the basis of predetermined saturation times^70^ (Supplementary Fig. 3). The samples were developed and fixed at 17 °C according to the manufacturer’s instruction (PHENISOL developer and HYPAM fixer, ILFORD). The cells were counterstained with 4′,6-diamidino-2-phenylindole (DAPI; final concentration, 2 µg ml^−1^) and enumerated under an epifluorescence microscope (Axio Imager M2, Carl Zeiss) equipped with the appropriate filter sets (filter sets 49 and 44, Carl Zeiss) and a camera (AxioCam MRm, Carl Zeiss). For each sample, more than 1,000 DAPI-stained cells in more than ten fields of view were examined. Microscopic images were analysed using Axio Vision SE64 Re4.9 (AxioVs40x64 V 4.9.1.0, Carl Zeiss) to count the DAPI-positive and DAPI-plus-CARD–FISH-positive cells and measure the area of the silver grain halo around the cells. DIC-positive cells were defined as those showing an autoradiographic signal above the background threshold, typically lower than 2–3 silver grains.

We measured the silver grain halo area around each tracer-positive cell, which can be converted to cell-specific uptake rates using corresponding bulk incorporation rates measured by scintillation counting^37^. The summed halo area of leucine-positive cells highly correlated with bulk ^3^H-leucine incorporation during three cruises (*R*^2^ = 0.89, *P* = 2.8 × 10^−12^, *n* = 23). For ^14^C-bicarbonate, the halo-to-bulk regression was strong in samples in the North Atlantic, (Geo; *R*^2^ = 0.89, *P* = 3.1 × 10^−6^, *n* = 11) but weak in the Pacific (SO248; *R*^2^ = 0.39, *P* = 0.018, *n* = 11), where detectable DIC-positive cell counts were relatively low. As an additional constraint, decompression is known to elevate heterotrophic activity in piezosensitive *Alteromonas*, so we applied count-based fractions for water-mass and global estimates instead, which were more stable and showed no significant postdecompression elevation^74^.

To increase the robustness under low cell availability, we pooled DIC-positive prokaryotic cells and DIC-positive *Alteromonas* cells across depths within the same water mass for each station (Extended Data Table 1) using the previous water-mass classification^75^. The fraction of DIC-assimilating cells identified as *Alteromonas* was then calculated as *P* = SUM_DICAlt_/*n*, where SUM_DICAlt_ is the pooled DIC-positive *Alteromonas* cells and *n* is the pooled DIC-positive prokaryotic cells. Only water masses with *n* ≥ 5 were retained for further analysis (Extended Data Table 1). Approximate 95% binomial CIs were calculated using the normal approximation *P* ± 1.96·√(*P*(1 − *P*)/*n*) and bounded to (0, 1) before converting to percentages. To estimate uncertainty in the overall mean *Alteromonas* contribution across all water masses (0–42.9%, median 16.7%), we used a nonparametric bootstrap (10,000 resamples of pooled percentages) and reported the 95% CI (9.9–28.3%) from the 2.5th and 97.5th percentiles of the bootstrap distribution.

For Sorokin’s calculation, depth-integrated dark DIC-fixation rates (µmolC m^−2^ d^−1^) were calculated for depths >250 m in the North Atlantic (Geo) and the North Pacific (SO248; northern hemisphere stations) using trapezoidal integration of volumetric fixation rates (µmolC m^−3^ d^−1^) over sampled depth intervals. Station-averaged fluxes (North Atlantic *n* = 6; North Pacific, *n* = 10) were extrapolated to sub-basin areas (North Atlantic: 4.15 × 10^7 ^km^2^, North Pacific: 7.70 × 10^7 ^km^2^, from ETOPO1), considering that 97% of the area lies below the epipelagic^38^. A 250-m cutoff was applied to avoid unsupported extrapolation across the highly variable ~200-m layer. The heterotrophic carbon demand (OC_need_, PgC yr^−1^) was estimated on the basis of the anaplerotic activity of heterotrophs, assuming that anaplerotic DIC fixation accounts for 10% of heterotrophic carbon biomass. This yields: OC_need_ = DICf_int_/0.10, where DICf_int_ (PgC yr^−1^) is the sub-basin-integrated dark DIC fixation.

### Statistical analysis

All analyses were conducted with customized Python, version 3.8.15, and Biopython (version 1.78)^76^. Statistics were performed and graphics developed using R (v4.1.1), GMT (v6.2.0) and ODV (v 5.7.1).

## Online content

Any methods, additional references, Nature Portfolio reporting summaries, source data, extended data, supplementary information, acknowledgements, peer review information; details of author contributions and competing interests; and statements of data and code availability are available at 10.1038/s41561-026-02013-1.

## Supplementary information

Supplementary Information Supplementary Discussion, Notes 1–2, Tables 1–4, Figs. 1–3 and references.

## Data Availability

All data are available in this Article and its Supplementary Information or in a public repository. Bulk dark DIC-fixation rates and associated raw DPM values are available via GitHub at https://github.com/chie-amano/cruise_data.

## Extended data

**Extended Data Table 1:** Percentage contribution of putative anaplerotic dissolved inorganic carbon (DIC) fixation by Alteromonas to the total dark DIC fixation in the Atlantic and Pacific Ocean

Region	Cruise	Station	WM	Depth (m)	Mean depth (m)	Total DAPI counts (cells)	DIC-positive Prok (cells)	DIC-positive Alt (cells)	% count contribution	% uptake contribution	Used in analysis (DIC-positive Prok: n ≥ 5)
Atlantic	Geo	6	ASUW	250	325	3544	44	10	22.7	46.9	yes
				400							
		11	WASIW	1250	1250	1659	8	3	37.5	41.3	yes
			NADW	1750	2583	5626	9	1	11.1	12.0	yes
				2500							
				3500							
		16	WNACW	250	375	3865	14	3	21.4	64.3	yes
				500							
			WASIW	1250	1250	1488	0	0	NA	NA	no
			NADW	2000	2500	3247	7	3	42.9	75.8	yes
				3000							
	Rad	11	EASIW	1800	1800	1277	19	1	5.3	2.9	yes
Pacific	SO248	6	PEW	200	367	10023	2	0	0.0	0.0	no
				400							
				500							
			AAIW	1000	1000	1343	0	0	NA	NA	no
			CDW	2000	2000	1127	1	0	0.0	0.0	no
		10	PSIW	500	750	3241	19	7	36.8	40.1	yes
				1000							
		14	PSUW	200	200	5383	21	0	0.0	0.0	yes
			PSIW	500	500	1829	6	0	0.0	0.0	yes
			PDW	1000	1500	2313	25	3	12.0	4.3	yes
				2000							

The table shows the proportion (%) of dark DIC-assimilating cells identified as *Alteromonas* within pooled water masses. Silver grain halo area from microautoradiography was recorded and converted to cell-specific DIC uptake (shown here as ‘% uptake contribution’), but these values were not used for global CO_2_ scaling due to low DIC-positive cell counts (for example, one station in SO248) and decompression-related activity elevation in piezosensitive *Alteromonas*. Count-based fractions were used for water-mass and global estimates instead, as they are less prone to decompression bias^74^.

Abbreviations: WM, water mass; Alt, Alteromonas; Prok: prokaryotes. Determination of the water masses follow in the previous study^75^: Atlantic Subarctic Upper Water (ASUW), Western North Atlantic Central Water (WNACW), Western Atlantic Subarctic Intermediate Water (WASIW), North Atlantic Deep Water (NADW), Eastern North Atlantic Subarctic Intermediate Water (EASIW), Pacific Equatorial Water (PEW), Antarctic Intermediate Water (AIW), Circumpolar Deep Water (CDW), Pacific Subarctic Upper Water (PSUW), Pacific Subarctic Intermediate Water (PSIW), Pacific Deep Water (PDW).

## Code availability

Scripts for the customized Python and Bipython are available via Zenodo at 10.5281/zenodo.7721930 (ref. ^77^).
