# Supplementary material for: Major contribution of anaplerosis to inorganic carbon fixation in the dark ocean
Source: Nat Geosci. 2026 Jun 15;19(8):938–44. doi: 10.1038/s41561-026-02013-1 (PMC13447088; doi:10.1038/s41561-026-02013-1)
Supplement: Supplementary file 1 [file 41561_2026_2013_MOESM1_ESM.pdf]

nature geoscience

Supplementary information https://doi.org/10.1038/s41561-026-02013-1

Major contribution of anaplerosis to inorganic carbon fixation in the dark ocean

In the format provided by the authors and unedited

Supplementary Discussion

Anaplerotic DIC fixation in *Alteromonas mediterranea* (AltDE) under controlled culture conditions.

AltDE exhibited clear differences in their growth depending on temperature and organic carbon source (Fig. 2a). Growth rate increased strongly with temperature (linear mixed-effects model: $F(1,29) = 273.3$, $p = 2.7 \times 10^{-16}$) and was consistently higher on Glc than on Ala ($F(1,29) = 741.1$, $p = 2.2 \times 10^{-16}$), showing more than 5-fold faster growth rates on Glc at both temperatures (growth rate: 0.04 vs. 0.21 h^{-1} at 13°C; 0.06 vs. 0.47 h^{-1} at 23°C).

DIC fixation occurred in all the treatments (Fig. 2b), confirming that the ^{14}C-bicarbonate was assimilated into AltDE biomass. Given that the AltDE genome lacks canonical autotrophic CO_2-fixation pathways but encodes and expresses anaplerotic carboxylases, this DIC assimilation is consistent with anaplerotic reactions that replenish central metabolic intermediates during heterotrophic growth. Although direct validation of *in vivo* flux would require future isotopic tracing experiments, this interpretation is in agreement with our flux balance analysis (Fig. 2c). Temperature and substrate type both significantly affect cell-specific DIC fixation, with higher rates at 23°C (nested ANOVA: $F(1,12) = 312.9$, $p = 5.8 \times 10^{-10}$) and consistently higher DIC fixation on Glc than Ala ($F(1,12) = 221.7$, $p = 4.2 \times 10^{-9}$).

Increasing glucose concentrations (from 50 to 500 µmol C L^{-1}), however, did not elevate cell-specific DIC fixation at 13°C (0.13 ± 0.01 vs 0.12 ± 0.01 fmol C $cell^{-1}$ h^{-1}; pairwise comparison from two-way ANOVA, $p = 0.40$, –8%), whereas at 23°C, it increased from 0.25 ± 0.01 to 0.31 ± 0.05 fmol C $cell^{-1}$ h^{-1}(corresponding to an increase of 24%, $p = 0.001$; Fig. 2b), indicating that substrate enrichment affected DIC fixation only under higher temperature conditions ($F(1,8)=17.5$, $p = 0.003$). In addition, in glucose treatments, temperature explained most of the variance in DIC fixation (partial $\eta^2 = 0.97$). These patterns indicate that carbon assimilation was kinetically limited by low temperature (at 13°C) rather than by substrate supply.

Consistent with cell-specific DIC fixation patterns, cell-specific heterotrophic biomass production was higher on Glc than Ala (Extended Data Fig. 3a; two-way ANOVA: $F(1,8) = 1014.6$, $p = 1.0 \times 10^{-9}$) and greater at 23°C than 13°C ($F(1,8) = 247.2$, $p = 2.7 \times 10^{-7}$), with strong positive correlation with DIC fixation ($R^2 = 0.88$, $p = 1.0 \times 10^{-8}$; Extended Data Fig. 3d). A similar trend was observed when applying microautoradiography (Extended Data Fig. 3e), showing that DIC assimilation correlates with heterotrophic activity. This relationship suggests a close coupling between heterotrophic DIC fixation and biomass production, consistent with its role in maintaining central metabolic flux during growth.

While modest in magnitude, the relative contribution of anaplerotic carbon fixation (% DICfix = cell-specific DIC fixation / cell-specific heterotrophic biomass production) differed significantly among treatments (nested ANOVA: temperature, $F(1,12) = 14.4$, $p = 0.003$; substrate, $F(1,12) = 38.2$, $p = 4.7 \times 10^{-5}$) and was generally higher when the treatment was amended with Ala than Glc at 23°C (post-hoc pairwise comparison, p=0.0002–0.003), while the two substrates had a comparable effect on anaplerotic carbon fixation at 13°C (p = 0.21–

0.99). This pattern aligns with previous findings that DIC fixation contributes a greater fraction of biomass carbon when cells utilize small carbon substrates (e.g., C_3 alanine) than larger carbon substrates (C_6 glucose)[2]. These results also indicate that although anaplerotic CO_2 fixation covaries with heterotrophic metabolism overall, its relative contribution to total carbon flow depends on substrate type and temperature.

At the same substrate concentration, glucose (C_6) provides twice as much carbon per mol for biomass production as alanine (C_3), which stoichiometrically explains the higher heterotrophic biomass production and DIC fixation observed in *Alteromonas* with glucose (Extended Data Fig. 3a, Fig. 2b). Glc enters central metabolism through glycolysis and the pentose phosphate pathway, directly providing energy and biosynthetic precursors (Fig. 2c). In contrast, when Ala is the sole carbon source, it is first converted to pyruvate through transamination, and part of this carbon is then routed through gluconeogenesis to regenerate sugar intermediates. This additional biosynthetic demand likely contributes to the lower growth, biomass production and greater anaplerotic carbon fixation observed under Ala relative to Glc. Flux balance modeling predicted that carbon flow through PEP carboxylase exceeded that through acetyl-CoA entry into the TCA cycle, and that the glyoxylate shunt contributed substantially to the TCA cycle (Fig. 2c). These results indicate that anaplerotic carbon fixation and the glyoxylate shunt jointly sustain the replenishment of the TCA cycle in AltDE. Furthermore, mapping the *Tara* Oceans metatranscriptome against AltDE genes encoding TCA-cycle enzymes further confirmed that anaplerotic and glyoxylate-shunt pathway genes were expressed in a wide range of oceanic regions (Extended Data Fig. 4).

Concerning respiration, the fraction of leucine respired by AltDE varied with temperature, but not with substrate type (Extended Data Fig. 3c; nested ANOVA, for temperature $F(1,12) = 204.7$, $p = 6.7 \times 10^{-9}$; for substrate $F(1,12) = 0.025$, $p = 0.88$). Respiration increased more strongly with temperature than biomass production (Extended Data Fig. 3a,b; paired Wilcoxon signed-rank, one-sided, $p = 0.0019$), yielding Q_{10} values in the order respiration > DIC fixation > biomass production across all treatments (Supplementary Table 2). Although DIC fixation also increased with temperature, it remained much smaller than respiratory carbon losses (Fig. 2c,d, Extended Data Fig. 3b). This pattern aligns with the reported decline in bacterial growth efficiency at higher temperatures, where respiration shows greater temperature sensitivity than biosynthesis[3-5]. Lower growth efficiency reflects increased respiratory carbon loss, which elevates the flux through oxidative decarboxylation reactions and increases the anaplerotic demand. The intermediate temperature sensitivity of DIC fixation is consistent with this need to replenish TCA cycle intermediates as their turnover accelerates. Yet, our ^{14}C-bicarbonate assays quantify only DIC retained in biomass, so they may underestimate total anaplerotic flux if some fixed DIC is rapidly respired.

Supplementary Notes

Supplementary Note 1. Peptide selection, phylogenetic reconstruction, and taxonomic assignment

From the reference sequences, a smaller subset of the peptides was selected by BLASTP at ≥70% alignment identity and a minimum bitscore ≥ 50 with any of OM-RGC.v2 peptides, after translation with Prodigal. Duplicate reference sequences were removed before the phylogenetic reconstruction with the reference sequences. If the number of reference sequences was too large then the number was reduced with CD-HIT[6], version 4.8.1, at a 95-99% identity threshold to obtain less than 1500 representative sequences. Representative phylogenetic trees for each predicted enzyme were constructed with IQ-TREE[7] version 2.1.4-beta, after alignment with MUSCLE[8] version 3.8.

In a first step to classify the sequences functionally and taxonomically, a first search was carried out by BLASTP of all translated metatranscriptome and metagenome sequences and a relaxed searching parameter (minimum bitscore of 50). The BLASTP database contained both the representative sets of peptides, with the proper label, and the rest of the peptides in the genome database. Since reference sequences might not contain all diversity of peptides from each gene, an HMM search was also carried out using the same HMMs models and the OM-RGC.v2 peptides. The subset of environmental peptides retrieved by BLASTP were placed on the reference phylogenetic trees to assign a taxonomic label. For the placement, OM-RGC.v2 peptides were aligned with the aligned reference sequences using the package PaPaRa[9] version 2.5. A maximum likelihood placement was carried out with EPA-ng[10], version 0.3.8, with an amino acid substitution model predicted with IQ-TREE. Finally, the jplace file was converted into a newick text file using gappa[11] version 0.7.1. Placed sequences with branch lengths longer than the longest distance between all pairs of sequences on the reference tree were removed (<1% of the sequences). Taxonomy assignments were given to each of the individual environmental peptide sequences on the basis of the placements in the jplace files and the GTDB classification of the reference genomes. When there were multiple placements in the jplace file, the sum of the 'like weight ratio' was first normalized with gappa (gappa edit filter–normalize-before) and the taxonomy was chosen as the lowest taxonomic rank that had a sum of 'like weight ratio' ≥80%. The taxonomic assigned and placement of the peptides were visually inspected with iToL[12] version 7. Further details and methods can be found in Baltar et al.[13]

Supplementary Note 2. AltDE culture experiment

<u>Leucine incorporation and leucine respiration</u>

For each of the triplicate cultures, 20 mL was distributed into three ~120 mL biological oxygen demand (BOD) flasks: one served as a formaldehyde-killed control, and the remaining two were used for live samples. Cultures were incubated for 2 h with ^{14}C-leucine (final concentration: 140 nM; L-^{14}C[U]-leucine, 250 mCi $mmol^{-1}$, ARC0656A, Hartman Analytic) following the determination of their kinetics (Supplementary Fig. 3). The BOD bottles were sealed with rubber stoppers with a plastic cup containing a chromatography paper (1Chr, 3001-

861, Whatman)[14]. Incubation was terminated by injecting 800 µL of 2N H_2SO_4. Immediately afterwards, 200 µL of phenethylamine was added onto the filter paper in the plastic cup. The BOD bottles were then placed on a shaker for 1h at room temperature. Thereafter, the filter papers were transferred to scintillation vials, the DPMs measured and the leucine respiration rates calculated based on the $^{14}CO_2$ released. For biomass production, water samples were filtered onto polycarbonate filters and the DPMs were counted with a scintillation counter similarly as described for the leucine incorporation measurements. This assay quantifies the distribution of ^{14}C-leucine between incorporation into biomass and release as $^{14}CO_2$, providing the fraction of substrate respired. It does not yield absolute respiration rates but enables comparison of carbon allocation under the same experimental conditions used for the ^{14}C-bicarbonate fixation measurements.

Cell abundance

Water samples (1 mL) were fixed with glutaraldehyde (0.5% final concentration) and stored at 4°C. If analysis could not be conducted within one day, the samples were flash-frozen in liquid N_2 for 15 min and stored at –80°C until further analysis. Prior to analysis, the frozen samples were thawed to room temperature. Prokaryotes were stained with SYBRGreen I in the dark for 10 min. Cell abundance was measured with an Accuri C6 Flow Cytometer (Accuri Cytometers Ltd) based on green fluorescence *versus* side scatter signature in a plot. Growth rates of AltDE were calculated from the cell counts obtained by flow cytometry (FCM) during the exponential phase using the formula: $\mu = \ln 2 / g$, where μ is growth rate (h^{-1}) and g is generation time (h). Growth rate was tested using a two-way mixed-effects model (*lme4*, *lmerTest*) with temperature and substrate as fixed factors and assay (^{14}C-bicarbonate and ^{14}C-leucine) as a random effect.

Amino acids concentration

The concentration of amino acids (AA) in AltDE cells was determined following a previous study[15]. Cells grown in Glc (50 µM C) and Ala (50 µM C) were collected onto polycarbonate filters (0.2 µm pore size, 25 mm diameter) and rinsed twice with borate buffer saline (0.1 M boric acid, 0.076M NaOH, 3.5% NaCl, pH 8). Duplicate filters were placed in 10 mL borate buffer saline, shaken for 1h and kept overnight at 4°C to resuspend the cells. The recovery rate after resuspension was determined by measuring cell abundance, yielding a 52% recovery. For protein analysis, the cells were hydrolyzed with 6M HCl at 110°C for 20 h after addition ascorbic acid (0.12 mM), and the concentrations of dissolved free amino acids were determined using a Shimadzu Nexera X2 ultra high-performance liquid chromatograph (UHPLC) with ortho-phthaldialdehyde (OPA) pre-column derivatization, as described in a previous study [16]. To account for amino acids destroyed during hydrolysis and not detectable by UHPLC, corrections were made using the relative abundance of amino acids based on the complete genome sequence of *Alteromonas*[17]. Blank correction was performed by measuring dissolved free amino acids in the hydrolyzed borate buffer saline. After correcting for non-measurable amino acids (27.8 % in this study), the relative gram percentage of leucine was 7.5 % in the Ala incubation and 7.9 % in the Glc incubation (Supplementary Table 3).

Cellular carbon content

Cells of AltDE incubated in Ala (50 µM C) and Glc (50 µM C) were collected onto pre-combusted GF/F filters (Whatman) using an acid-washed and combusted glass filtration system to determine particulate organic carbon (POC) concentrations. The filters were stored at –20°C until analysis. POC concentrations of the triplicate filters from each incubation condition were measured with an elemental analyzer (Vario Micro Cube, Elementar). To calculate per-cell carbon content, cell abundance in the water samples before filtration and in the filtrate was measured using FCM.

Calculation of heterotrophic biomass production and DIC fixation

For the AltDE culture experiments, empirical leucine to carbon conversion factors (CF_{leu}) for Ala and Glc incubations (both at 50 µM C and 23°C) were determined, according to the previous study[15]. The CF_{leu} values were calculated using the percentage of leucine, protein content, and carbon content in AltDE cells, resulting in 1.0 ± 0.1 and 1.1 ± 0.1 kg C per mol leucine (mean ± s.d., error propagated) for Ala and Glc, respectively (Supplementary Table 4). Isotope dilution (ID) in glucose treatments was ~1 (0.9 ± 0.3, mean ± s.d., n = 12) and the concentration of ^{3}H-leucine added was ~7 times higher than the leucine concentration in AltDE cells, making ID negligible. Thus, an ID value of 1 was applied.

Cell-specific heterotrophic biomass production (mol C cell^{-1} h^{-1}) was determined using cell abundance at the start (T_1) and end (T_2) of the assays within the exponential phase, leucine incorporation between T_1 and T_2 (mol Leu L^{-1}) and the empirical CF_{leu} values. Microautoradiography confirmed that nearly 100% of the cells were taking up the tracers. Cell-specific heterotrophic biomass production constant (k, mol cell^{-1} h^{-1}) during the duration of incubation in the exponential phase is calculated as: $k = \mu W / (N_2 - N_1)$, where W is production (mol C L^{-1}), N_1 and N_2 are cell abundance at T_1 and T_2, respectively, μ (= ln2 / g) is growth rate (h^{-1}). Similarly, cell-specific dark DIC fixation rates (mol C cell^{-1} h^{-1}) were calculated using the bulk dark DIC fixation rate (mol C L^{-1} h^{-1}) and cell abundance at T_1 and T_2. Cell-specific DIC fixation and production rates were analyzed by two-way ANOVA, and significant interactions were examined with *emmeans* (Tukey-adjusted post hoc tests). Q_{10} values were calculated by dividing the cell-specific DIC fixation or heterotrophic biomass production at 23°C by those at 13°C.

Supplementary Table 1. Media composition used as model input with alanine or glucose as the sole organic carbon source.

compounds	name	formula	minFlux	maxFlux	concentration
cpd00149	Co2+	Co	-100	100	0.001
cpd00099	Cl-	Cl	-100	100	0.001
cpd00067	H+	H	-100	100	0.001
cpd00063	Ca2+	Ca	-100	100	0.001
cpd00058	Cu2+	Cu	-100	100	0.001
cpd00048	Sulfate	SO4	-100	100	0.001
cpd00034	Zn2+	Zn	-100	100	0.001
cpd00030	Mn2+	Mn	-100	100	0.001
cpd00013	NH3	NH4	-100	100	0.001
cpd00009	Phosphate	HPO4	-100	100	0.001
cpd00001	H2O	H2O	-100	100	0.001
cpd00205	K+	K	-100	100	0.001
cpd00254	Mg	Mg	-100	100	0.001
cpd00971	Na+	Na	-100	100	0.001
cpd10515	Fe2+	Fe	-100	100	0.001
cpd10516	fe3	Fe	-100	100	0.001
cpd11574	Molybdate	H2MoO4	-100	100	0.001
cpd00244	Ni2+	Ni	-100	100	0.001
cpd00035	L-Alanine	C3H7NO2	-100	5	0.001
cpd00215	Pyridoxal	C8H9NO3	-100	5	0.001
cpd00104	BIOT	C10H15N2O3S	-100	5	0.001
cpd00305	Thiamin	C12H17N4OS	-100	5	0.001
cpd00393	Folate	C19H17N7O6	-100	5	0.001
cpd00644	PAN	C9H16NO5	-100	5	0.001
cpd00220	Riboflavin	C17H19N4O6	-100	5	0.001
cpd00218	Niacin	C6H4NO2	-100	5	0.001
cpd00635	Cbl	C62H88CoN13O14P	-100	5	0.001
cpd00557	Siroheme	C42H36FeN4O16	-100	5	0.001
cpd00028	Heme	C34H30FeN4O4	-100	5	0.001
cpd00007	O2	O2	-100	100	0.001
cpd00011	CO2	CO2	-100	100	0,001

The glucose medium for the metabolic model contained the following instead of alanine:

cpd00027	D-Glucose	C6H12O6	-100	5	0.001

Supplementary Table 2.

Q_{10} values for anaplerotic DIC fixation, heterotrophic biomass production, and leucine respiration by AltDE. The Q_{10} values were calculated from cell-specific rates measured at 13°C and 23°C. Values are presented as mean and s.d. (n = 3).

**C-source**	**DICfix**		**Biomass production**		**Leu resp.**	
	**Mean**	**s.d.**	**Mean**	**s.d.**	**Mean**	**s.d.**
Ala 50 µM C	2.1	0.4	1.3	0.1	2.9	0.2
Glc 50 µM C	1.9	0.1	1.7	0.1	3.3	0.7
Glc 500 µM C	2.6	0.3	2.4	0.3	4.1	1.0

Supplementary Table 3. Amino acid composition of AltDE proteins during the exponential phase under alanine and glucose incubations. The table lists the amino acid percentages in the proteins, with values corrected based on the complete genome sequence of *Alteromonas macleodii*. Corrected percentages are shown as g% corr.

AA	MW	*A. macleodii**		This study					
		mol%	g%	mol%		g%		g% corr.	
				Ala	Glc	Ala	Glc	Ala	Glc
Asparagine [a]	132.1	4.4	4.5	11.7 ± 0.0	13.5 ± 0.8	12.3 ± 0.0	14.2 ± 0.8	8.9 ± 0.0	10.3 ± 0.6
Aspartic acid [a]	133.1	5.4	5.6						
Glutamine [b]	146.2	4.1	4.6	27.2 ± 0.2	22.2 ± 0.2	31.4± 0.3	26.0 ± 0.3	22.7 ± 0.2	18.8 ± 0.2
Glutamic acid [b]	147.1	6.3	7.2						
Serine	105.1	6.8	5.6	6.1 ± 0.5	6.1 ± 1.4	5.0 ± 0.4	5.1 ± 1.2	3.6 ± 0.3	3.6 ± 0.8
Histidine [c]	155.2	2.3	2.7	4.1 ± 0.1	5.0 ± 0.4	5.0 ± 0.1	6.1 ± 0.5	3.6 ± 0.1	4.4 ± 0.4
Threonine [c]	119.1	5.5	5.1						
Glycine	75.1	6.5	3.8	13.8 ± 0.8	13.5 ± 1.7	8.1 ± 0.5	8.1 ± 1.0	5.9 ± 0.4	5.8 ± 0.7
Alanine	89.1	8.9	6.1	11.3 ± 0.4	13.9 ± 1.5	7.9 ± 0.2	9.8 ± 1.0	5.7 ± 0.2	7.1 ± 0.8
Arginine	174.2	4.5	6.1	5.0 ± 0.1	5.3 ± 0.0	6.9 ± 0.2	7.4 ± 0.1	5.0 ± 0.1	5.3 ± 0.0
Tyrosine	181.2	3.0	4.2	2.7 ± 0.0	2.9 ± 0.0	3.8 ± 0.0	4.1 ± 0.0	2.8 ± 0.0	3.0 ± 0.0
Valine	117.1	7.1	6.5	ND					
Phenylalanine	165.2	4.4	5.6	ND					
Isoleucine	131.2	6.1	6.2	ND					
Leucine	131.2	10.0	10.2	10.1 ± 0.5	10.5 ± 0.2	10.4 ± 0.5	11.0 ± 0.2	7.5 ± 0.4	7.9 ± 0.1
Cysteine	121.2	1.1	1.0	ND					
Lysine	146.2	5.8	6.6	8.0 ± 0.4	7.2 ± 0.0	9.1 ± 0.5	8.3 ± 0.0	6.6 ± 0.3	6.0 ± 0.0
Methionine	149.2	2.8	3.2	ND					
Proline	115.1	3.8	3.4	ND					
Tryptophan	204.2	1.2	1.9	ND					

*Amino acid percentages from a complete genome sequence of *A. macleodii* [17].

[a] sum of asparagine and aspartic acid in this study.

[b] sum of glutamine and glutamic acid in this study.

[c] sum of histidine and threonine in this study.

Values are shown as: mean ± |mean – replicate| (n = 2).

AA: amino acids; MW: molecular weight; Glc: Glc 50 μM C, Ala: Ala 50 μM C both at 23°C; ND: not determined.

Supplementary Table 4. Empirical leucine to carbon conversion factors (CF_{leu}) for AltDE in incubations with alanine (Ala) or glucose (Glc).

	Ala	Glc
Leu (g%)	7.5 ± 0.4 (2)	7.9 ± 0.1 (2)
Protein (fg $cell^{-1}$)	87.7 ± 0.0 (2)	103.2 ± 3.3 (2)
Carbon (fg $cell^{-1}$)	51.8 ± 4.6 (3)	69.6 ± 8.0 (3)
CF_{Leu} (kg C [mol leu]$^{-1}$)	1.0 ± 0.1	1.1 ± 0.1

Values are shown as mean ± s.d. (n = 3) or mean ± |mean – replicate| (n = 2). Sample sizes are indicated in the brackets. Error propagated for CF_{leu}. Isotope dilution applied as 1.

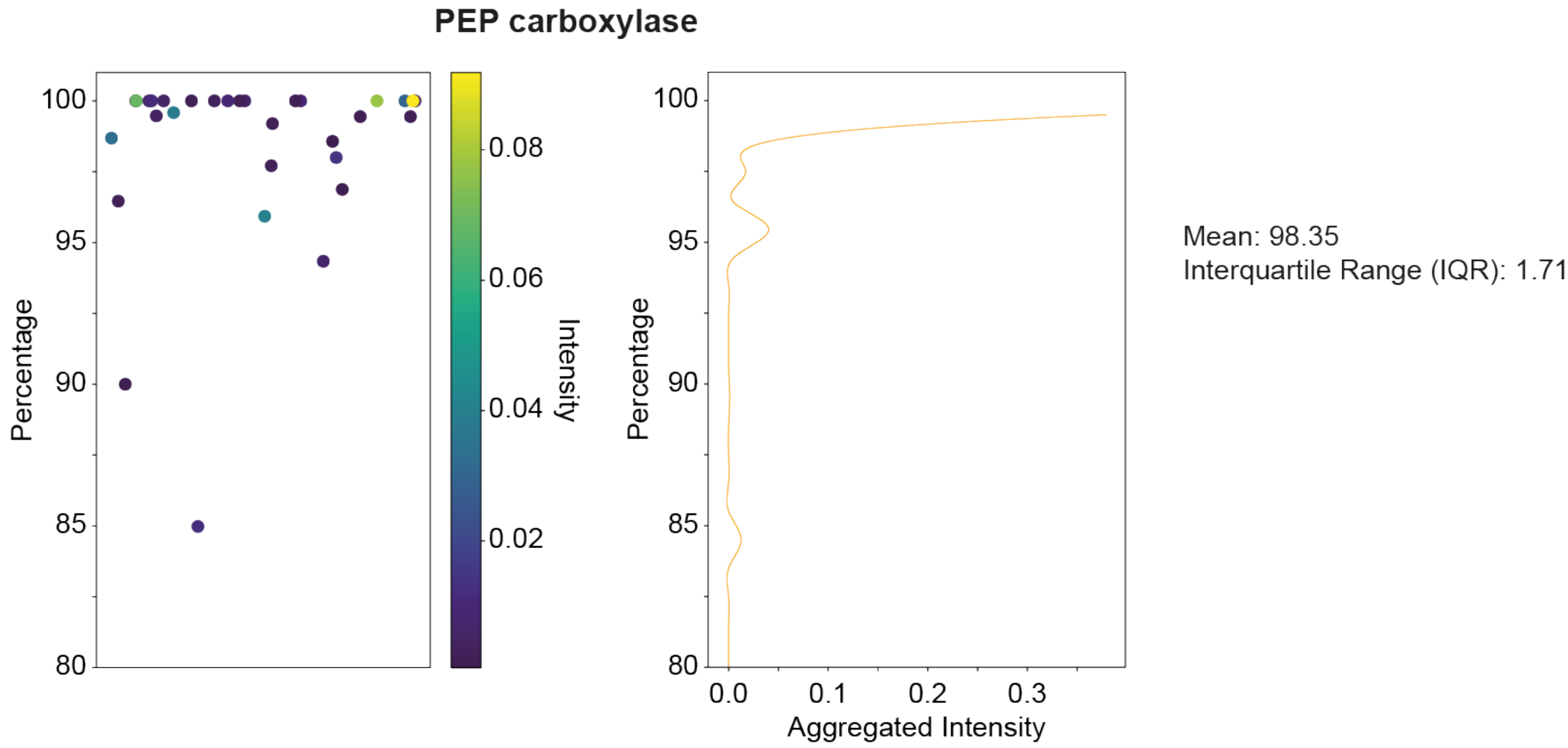

Supplementary Fig. 1. Contribution of Alteromonas peptides to overall sample proteome. Classified Alteromonas peptides, as described in Material and Method section of the main text, are represented in this plot by dots with their percent identity to the closest peptide in the genome database, classified with the GTDB Toolkit (GTDB-Tk) version 2.3.2 and database release 214[18]. The dots are jittered along the x-axis for better visualization. Color represents the average contribution of each peptide to the samples in the metaproteomic study by Zhao et al. [19] using their table of abundances associated to each peptide and sample, as described in their study. To the right, the panel shows the sum of the contribution of each peptide in intervals of 1% identity.

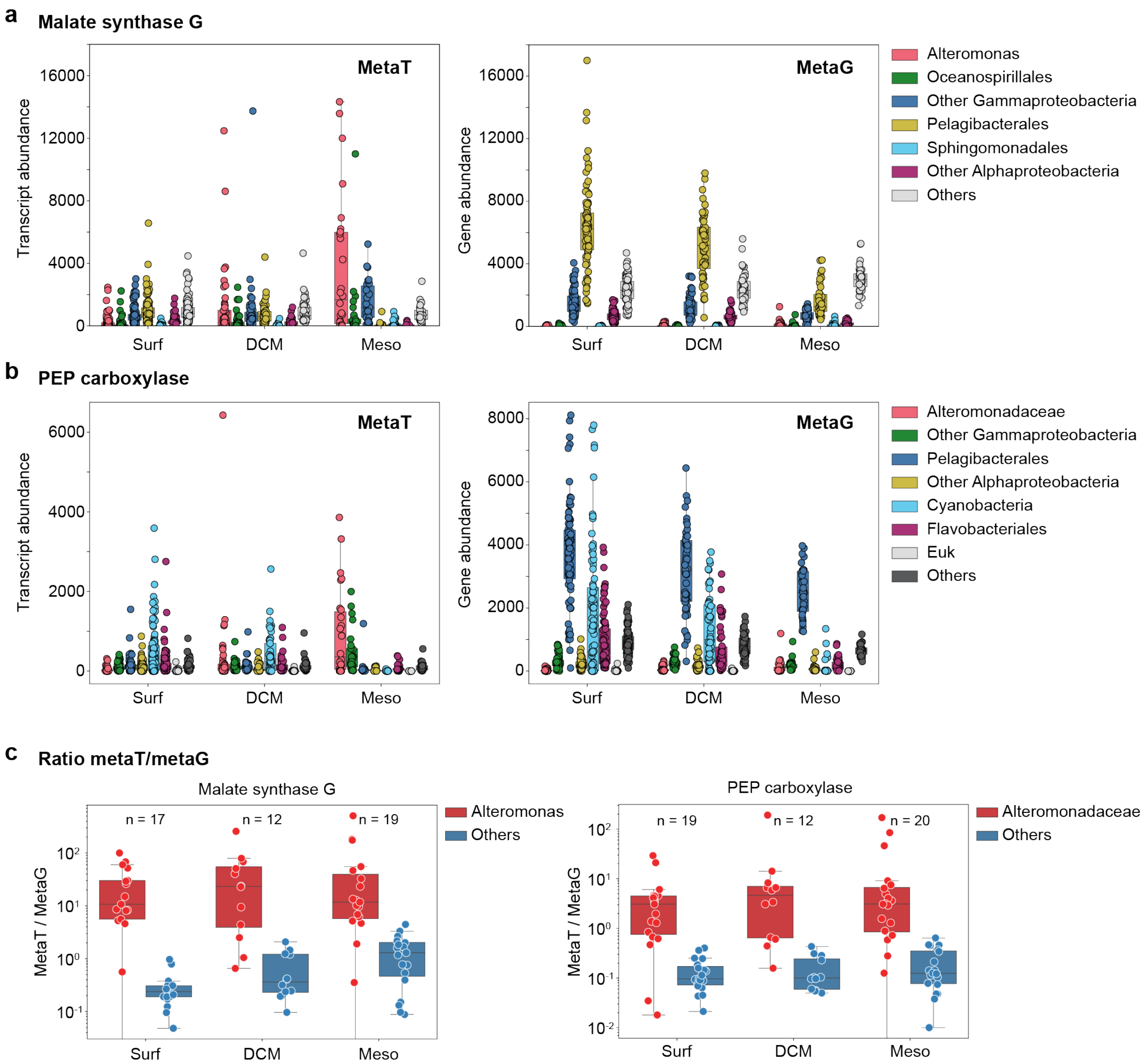

Supplementary Fig. 2.

Quantification method for gene abundance. (**a, b**) Anaplerotic gene expression from *Tara* Oceans metatranscriptome (metaT) and metagenomic (metaG), based on annotations and taxonomic assignment from the previous study [20], which uses a different annotation method from our study. These results are derived from the same dataset we used in our Fig. 1 and Extended Data Fig. 1, but they were calculated following a different approach to assign a function and a taxonomic label to the Microbial Reference Gene Catalog (OM-RGC.v2). We used our approach to increase the taxonomic resolution of our results. For instance, in this plot, since there were no PEP carboxylase peptides classified at the level of the genus (*Alteromonas* spp.), it is only possible to look at the family level (Alteromonadaceae). The genus *Alteromonas* spp. (when available) or the family *Alteromonas* spp. dominate the expression of the same anaplerotic genes as we found in our Fig. 1 and Extended Data Fig. 1, confirming the validity of our analyses. (**c**) Ratio of metatranscriptome to metagenome for the 51 samples where both metatranscriptomes and metagenomes are available in the previous study [20], showing that *Alteromonas* and/or Alteromonadaceae are more active than the other prokaryotic taxa. Boxes represent the interquartile range (Q1–Q3), with the central line indicating the median; whiskers denote the minimum and maximum values.

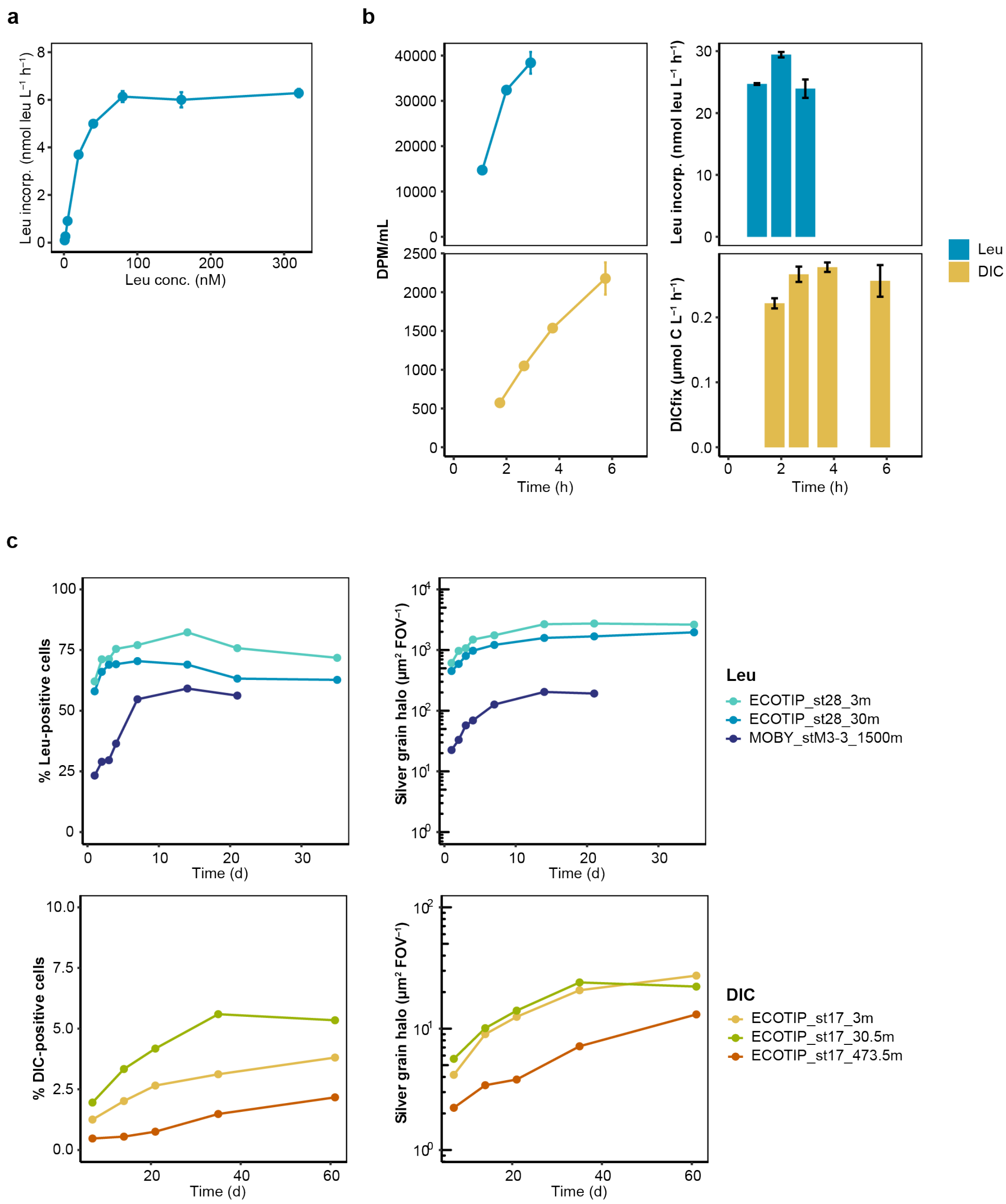

Supplementary Fig. 3.

Methodological adjustments and kinetics. (**a**) Concentration kinetics for AltDE cultures to determine the saturation concentration of tracer leucine, resulting in a final concentration of 140 nM. AltDE was incubated in Glc (50 µM C) at 23°C. (**b**) Duration kinetics for leucine incorporation (Leu incorp.) and dark DIC fixation (DICfix) rates, with optimal incubation times of 2 hours for leucine and 4 hours for DIC. (**c**) Detection of leucine-positive (upper panels) and DIC-positive (lower panels) cells under varying exposure times for microautoradiography. The left panels show the relative abundance of tracer-positive cells, while the right panels display the total silver grain halo sizes per field of view (FOV) of the microscope. Natural communities from stations at 69.1267°N, 52.8072°W (ECOTIP_st17), 70.410°N, 56.2697°W (ECOTIP_st28), and 68.0523°E, 50.7001°S (MOBY_stM3-3) were incubated with either ^{3}H-leucine or ^{14}C-bicarbonate (see Methods). Depths are indicated in the legends. FOV: field of view. Points and bars represent mean values; error bars indicate ± s.d. of n = 3.

Supplementary References

1 Sorokin, J. I. On the carbon dioxide uptake during the cell synthesis by microorganisms. *Z Allg Mikrobiol* **6**, 69-73 (1966).

2 Braun, A. *et al.* Reviews and syntheses: Heterotrophic fixation of inorganic carbon - significant but invisible flux in environmental carbon cycling. *Biogeosciences* **18**, 3689-3700 (2021).

3 Rivkin, R. B. & Legendre, L. Biogenic carbon cycling in the upper ocean: effects of microbial respiration. *Science* **291**, 2398-2400 (2001).

4 Apple, J. K., Giorgio, P. & Kemp, W. M. Temperature regulation of bacterial production, respiration, and growth efficiency in a temperate salt-marsh estuary. *Aquat Microb Ecol* **43**, 243-254 (2006).

5 Lonborg, C., Baltar, F., Carreira, C. & Moran, X. A. G. Dissolved Organic Carbon Source Influences Tropical Coastal Heterotrophic Bacterioplankton Response to Experimental Warming. *Front Microbiol* **10**, 2807 (2019).

6 Fu, L., Niu, B., Zhu, Z., Wu, S. & Li, W. CD-HIT: accelerated for clustering the next-generation sequencing data. *Bioinformatics* **28**, 3150-3152 (2012).

7 Nguyen, L.-T., Schmidt, H. A., von Haeseler, A. & Minh, B. Q. IQ-TREE: A fast and effective stochastic algorithm for estimating maximum-likelihood phylogenies. *Mol Biol Evol* **32**, 268-274 (2015).

8 Edgar, R. C. Muscle5: High-accuracy alignment ensembles enable unbiased assessments of sequence homology and phylogeny. *Nat Commun* **13**, 6968 (2022).

9 Berger, S. A. & Stamatakis, A. Aligning short reads to reference alignments and trees. *Bioinformatics* **27**, 2068-2075 (2011).

10 Barbera, P. *et al.* EPA-ng: Massively Parallel Evolutionary Placement of Genetic Sequences. *Syst Biol* **68**, 365-369 (2019).

11 Czech, L., Barbera, P. & Stamatakis, A. Genesis and Gappa: processing, analyzing and visualizing phylogenetic (placement) data. *Bioinformatics* **36**, 3263-3265 (2020).

12 Letunic, I. & Bork, P. Interactive Tree Of Life (iTOL) v5: an online tool for phylogenetic tree display and annotation. *Nucleic Acids Res* **49**, W293-W296 (2021).

13 Baltar, F. *et al.* A ubiquitous gammaproteobacterial clade dominates expression of sulfur oxidation genes across the mesopelagic ocean. *Nat Microbiol* (2023).

14 Clifford, E. L. *et al.* Taurine is a major carbon and energy source for marine prokaryotes in the North Atlantic Ocean off the Iberian Peninsula. *Microb Ecol* **78**, 299-312 (2019).

15 Simon, M. & Azam, F. Protein content and protein synthesis rates of planktonic marine bacteria. *Mar Ecol Prog Ser* **51**, 201–213 (1989).

16 Tinta, T. *et al.* Microbial processing of jellyfish detritus in the ocean. *Front Microbiol* **11**, 590995 (2020).

17 Moura, A., Savageau, M. A. & Alves, R. Relative amino acid composition signatures of organisms and environments. *PloS one* **8**, e77319 (2013).

18 Chaumeil, P. A., Mussig, A. J., Hugenholtz, P. & Parks, D. H. GTDB-Tk: a toolkit to classify genomes with the Genome Taxonomy Database. *Bioinformatics* **36**, 1925–1927 (2020).

19 Zhao, Z. *et al.* Metaproteomic analysis decodes trophic interactions of microorganisms in the dark ocean. *Nat Commun* **15**, 6411 (2024).

20 Salazar, G. *et al.* Gene expression changes and community turnover differentially shape the global ocean metatranscriptome. *Cell* **179**, 1068-1083.e1021 (2019).
